# Acidosis, Iron Dyshomeostasis and Inflammatory Injury

**DOI:** 10.3390/ijms27073279

**Published:** 2026-04-04

**Authors:** Rosaria Ingrassia, Andrew J. Ghio, Michael Garrick

**Affiliations:** 1 Section of Biotechnologies, Department of Molecular and Translational Medicine, University of Brescia, 25123 Brescia, Italy; 2 US EPA, Chapel Hill, NC 27599, USA; ghio.andy@epa.gov; 3 Emeritus of Biochemistry & Pediatrics, University at Buffalo, Buffalo, NY 14260, USA; mgarrick@buffalo.edu

**Keywords:** pH conditioning, divalent metal transporter 1 (DMT1), transferrin (Tf), non-transferrin bound iron (NTBI), iron metabolism, iron chelation, acidosis, inflammation, metal transport, neurodegeneration

## Abstract

Normal steps in uptake of non-heme iron by the gastrointestinal tract include ferrireduction and import across the apical enterocyte membrane by divalent metal transporter 1 (DMT1), responsible for the uptake of non-transferrin bound iron (NTBI). This metal import by the intestinal epithelium requires an acidic milieu generated by the proton pump H(+)/K(+) ATPase (ATP4). Gastrointestinal uptake of metal can be affected by altering the acid milieu (e.g., proton pump inhibitors). After metal uptake by enterocytes, ferroxidation and export of the metal by ferroportin (FPN) at the basolateral membrane leads to the export of iron bound to transferrin (Tf). In peripheral tissues, cellular uptake of circulating iron is mediated by receptor-mediated endocytosis of Tf-bound iron, with DMT1 transporting the metal out of the endosomal compartment under acidic conditions generated by the vacuolar H^+^-ATPase. Acidosis is frequently associated with inflammation. The two derangements have relevant consequences like improved solubilization of iron, increased expression of Dmt1, elevated Fe^2+^ uptake due to DMT1’s ability to cotransport H^+^, dissociation of Fe-Tf and hepcidin decreasing Fe export via FPN. These changes result in intracellular iron sequestration that frequently becomes noxious. Pharmacological strategies to inhibit NTBI transport are proposed to protect against iron overload associated with acidosis and inflammation.

## 1. Introduction

**Figure 1:**
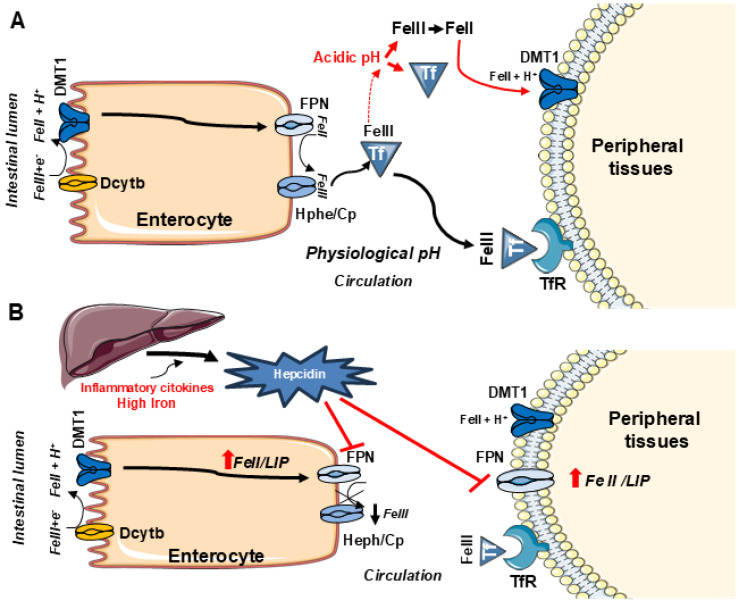
Iron absorption in acidic environments. An enterocyte is represented on the left, while a cell in a peripheral tissue is shown on the right. (**A**) The acidic milieu drives FeII entry via DMT1 into the enterocyte from which FeIII emerges bound to Tf and exits through FPN after oxidation by Cp (shown here) or Hp. The pathway for delivery to peripheral tissue involves FeIII-Tf-TfR. Acidic conditions, such as those observed during inflammation, favor greater NTBI import via DMT1. (**B**) Systemic hepcidin upregulation, consequent to inflammation and iron overload, downregulates FPN and inhibits intracellular iron export with consequent increase in the cytosolic Labile Iron Pool (LIP).

Iron is an essential element in cellular homeostasis linked to metabolic functions throughout a healthy human life. Normal iron homeostasis requires a dynamic equilibrium between systemic iron metabolism and distribution to essential intracellular sites. In duodenal enterocytes (Figure 1A), dietary non-heme iron crosses the apical membrane in an acidic milieu via the proton-coupled importer of iron, divalent metal transporter 1 (DMT1) (also known as solute carrier family 11 member 2 (SLC11A2), natural resistance-associated macrophage protein 2 (NRAMP 2), and divalent cation transporter-1 (DCT1)) [1,2]. After uptake, iron transits the cell aided by a chaperone [3,4], exiting the basal membrane while being oxidized to the ferric form by hephaestin (Hp) or ceruloplasmin (Cp). The metal is then transported from the enterocyte via the only known cellular iron exporter, ferroportin (FPN) (also known as SLC40A1 and metal transporter protein-1 (MTP1)) [5,6,7]. This export of iron from the enterocyte results in the metal being bound by transferrin (Tf), the circulating transport protein. Iron transported to the peripheral tissues is largely Tf-bound [8].

**Figure 2:**
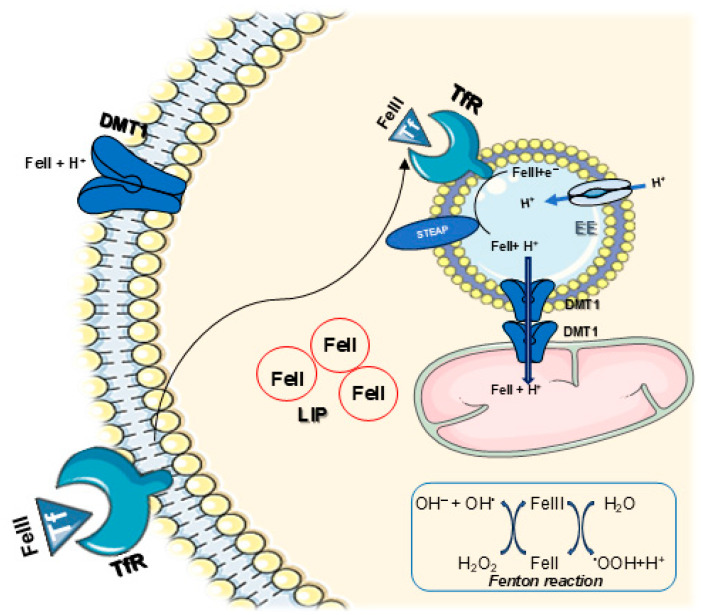
An endosome and a mitochondrion interact via ‘kiss and run’ [16]. The vacuolar ATPase drives protons into the endosome (near top) while DMT1 on both the endosomal membrane and on the outer mitochondrial membrane contacts sufficiently to enable the increased Fe^2+^ uptake from the vesicle to the mitochondria by an inward-directed proton gradient.

In most cells, transferrin receptor-mediated endocytosis of Tf is the principal iron delivery mechanism. This internalization activates the Tf cycle through which the metal-bound Tf is imported from the blood plasma (Figure 1A) at the cell surface by transmembrane glycoprotein receptors (TfR1 and TfR2) via clathrin-mediated endocytosis with endosome formation. Endosome acidification by a proton pump drives the release of Tf-bound metal; DMT1 then transports the iron out of the endosome. The apo-Tf/Tf receptor complex in the endosome moves to the cell surface where apo-Tf is released back into the bloodstream. During this Tf cycle, protons participate with DMT1 in the release and chemical reduction of iron through acidification, after the import across the endosomal membrane; then iron is released to pivotal sites including mitochondria [9,10,11,12] (Figure 2). Close contact between DMT1 on the endosome and mitochondrion can aid this process [13]. Cellular uptake of Tf-bound iron is tightly regulated in normal conditions to limit excessive assimilation and prevent overload, through IRE/IRP-dependent post-translational regulation in response to changes in the intracellular iron concentrations [14,15].

DMT1, expressed in four isoforms and transporting up to eight metals, is also responsible for a substantial proportion of non-transferrin bound iron (NTBI) transport (large red arrow, Figure 1), in addition to its involvement in the Tf cycle [17]. With excessive gastrointestinal iron uptake (e.g., hereditary hemochromatosis), there can be increased concentrations of NTBI entering the plasma. This metal is possibly redox-active and is ordinarily rapidly cleared from plasma. NTBI, however, can lead to overload if elevations persist causing pathological changes in peripheral tissues [8]. In addition to DMT1 and Tf/TfR, the regulation of the iron exporter FPN contributes to metal homeostasis. FPN levels at the cell membrane are influenced by its post-translational degradation, controlled by the inflammation-dependent systemic regulation of hepcidin (HAMP) (Figure 1B) [5]. With increased availability of this liver-derived peptide hormone (e.g., during inflammatory injury), FPN expression is downregulated at the cell membrane. Consequently, iron release from the enterocyte with translocation to Tf/TfR and peripheral tissues does not occur with decreased FPN availability [18]. Very recently, a French group [19] confirmed and extended earlier reports [20,21] that hepcidin also directly regulates duodenal DMT1, evaluated in terms of post-translational regulation with focus at (−)/(+)IRE isoforms, which was not significantly altered. The latest paper also includes a commentary [22] that helps readers appreciate the strength of the evidence that hepcidin directly regulates both iron influx and iron efflux in enterocytes as a response to inflammation and or iron overload. The French group’s evidence also points to post-transcriptional regulation of DMT1 by hepcidin, leading one to speculate that the regulation is primarily post-translational as for FPN. They apparently address DMT1 isoforms only at the mRNA level and not at the protein level; this circumstance raises issues discussed under Section 4.1 and Section 5 below.

## 2. Acid-Sensing Molecules

DMT1 exhibits maximal uptake of divalent metals, including iron, in acidic conditions, as observed in HEK293 cells and Caco2 cells [17,23,24,25,26,27]. Although the data can look like a pH optimum, the relationship of DMT1 activity with pH reflects proton co-transport with Fe^2+^, as originally postulated [1,28]. Lower pH values may also induce the chemical reduction of ferric iron. Hence, the gastrointestinal cytochrome B reductase (DCYTB), present on the enterocytic apical membrane, operates efficiently in the acidic milieu and catalyzes the reduction of ferric to ferrous ion, the substrate for DMT1 uptake at the same surface [29]. Interactions between acidosis and iron metabolism have been evaluated in terms of metal absorption. Import requires an acidic environment generated by the activity of the proton pump H(+)/K(+) ATPase (ATP4), expressed on the gastric parietal cells [30]. ATP4 is upregulated by hepcidin, the iron regulatory peptide induced by pro-inflammatory cytokines during the systemic inflammatory response and by elevated iron levels with a consequent increase in gastric acidity, thus supporting an in vivo association between iron metabolism and acidosis. With acidosis, liver mRNA and serum levels of hepcidin increased. These changes accompany downregulation of both mRNA and protein levels of intestinal DMT1 and FPN and significantly increased serum ferritin. Increasing dietary iron impacts ATP4A protein expression as well as serum, hepatic, and gastric hepcidin. In further support of a relationship between this proton pump ATPase activity and iron homeostasis, hepcidin knockout (KO) mice show a significant lowering of ATP4A expression, and in wild-type mice downregulated hepcidin expression levels were activated by pantoprazole while being downregulated by histamine, thus defining a reciprocal link between acidic pH and metal balance [31].

The acidic environment of the intestine promotes iron absorption by supporting the reduction of insoluble iron (Fe^3+^) to soluble iron (Fe^2+^) ions and permitting the formation of soluble chelates [32]. Proton pump inhibitors (e.g., omeprazole) reduce gastrointestinal acid production by inhibiting the H^+^/K^+^ ATPase proton pump and have been used for decades to treat acid-related disorders. Clinical studies have demonstrated that iron deficiency is frequently induced in patients with long term use of proton pump inhibitors [33,34].

Cells respond to acidic conditions with alterations in iron homeostasis [35]. During the Tf cycle, an acidic environment develops in the endosome via the action of a vacuolar H^+^-ATPase [36,37] with an additional possible contribution by DMT1 [10]. Although multiple regulatory mechanisms contribute to overall cellular pH, the vacuolar H^+^-ATPase is the primary regulator of pH in organelles [38]. The vacuolar H^+^-ATPase is an ubiquitous proton pump that couples ATP hydrolysis with proton translocation across the membranes of vacuoles/lysosomes, endosomes, the Golgi apparatus, and secretory vesicles and, in some cells, the plasma membrane. The acidic environment that results aids in the release of Fe^3+^ from Tf [10]. A membrane-associated ferric reductase analogous to DCYTB, called STEAP3 (Six-Transmembrane Epithelial Antigen of the Prostate 3) [35,36,39,40] catalyzes the chemical reduction of Fe^3+^ to Fe^2+^. The solubility of Fe^2+^ is greater in more acidic environments and both DCYTB and STEAP3 perform optimally in such a milieu. While loss of the vacuolar H^+^-ATPase is lethal in higher organisms, the loss of such function in unicellular organisms results in organelle acidification defects, chronic oxidative stress, and the disruption of iron homeostasis [38]. Indeed, the v-ATPase is a universal proton pump devoted to the control of intracellular pH homeostasis and entry of pathogen and toxins through the endosomal compartments, via protein degradation, in various cell organelles in almost all eukaryotic organisms, thus being responsible for both physiological and pathological conditions [41].

Other proteins are involved in maintaining tissue and cellular pH through electrolytic equilibria, such as the acid-sensing ion channels (ASICs) and the Carbonic Anhydrases (CaAs). These have been described in cancer environments and recently studied as activators of neurodegeneration. ASICs are a family of molecules predominantly expressed in the peripheral and central nervous system (CNS) [42]. They act as potent proton sensors to detect extracellular acidification in the periphery and brain during injury, inflammation, ischemia, epilepsy, and tumors [43]. The ASIC1a isoform seems to be involved in several neurodegenerative and neuroinflammatory pathologies, as well as in inflammatory pain [43].

Normal neuronal activity requires the maintenance of cytosolic pH in the physiological range, whereas deviations yield pathological conditions with potentially serious consequences [44]. In pathological conditions such as inflammation, ischemic strokes, and seizures that accompany several neurodegenerative diseases, pH drops and acidification can correlate with hyperexcitability [45]. ASIC1a channels, acting as proton sensors, are highly enriched in brain neurons and activated by acidosis during ischemia [46]. Mammalian cardiomyocytes express ASIC subunits in both plasma membranes and intracellular compartments. The ASIC2a/3 channel is involved as a molecular sensor of cardiac ischemic pain with pH-dependent activation [47]. Acidosis exacerbates ischemic injury and significantly affects tissue function. A study of ASIC1 variants [48] shows they are associated with ischemic injuries (e.g., cardiac and cerebrovascular). Specific pharmacological inhibitors of ASIC1a have a protective effect [48].

Carbonic Anhydrase (CaA) isoforms are critical in pH homeostasis through their catalysis of the hydration of CO_2_ to produce bicarbonate and a proton [49]. This family of zinc metalloenzymes participates in physiological processes such as pH and iron homeostasis, carbon dioxide transport, cerebrospinal fluid formation and turnover. Neurovascular dysfunction is an important determinant in the pathogenesis of both Alzheimer’s disease (AD) and stroke. Upregulation of CaAs in extracellular acidification becomes significant particularly in tumor environments. CaAs may also contribute to the development of cerebrovascular injury, as reported in a study of FDA-approved CaA modulators [50]. CaA inhibitors (CAIs) increase the activity of antioxidant enzymes such as superoxide dismutase-1 and heme-oxygenase-1, with positive results in cognitive performance in mouse models of amyloidosis. In addition, CAIs not only block caspase-9 activation and apoptosis caused by amyloid beta (Aβ) in cerebrovascular endothelial cells but also increase the protective activation of nuclear factor erythroid 2-related factor 2 (Nrf2) in cortical neuron models challenged by Aβ in vitro. This evidence supports the hypothesis that CAIs may exert a protective role in pathological neurovascular events and in models of amyloidosis [50], tested in clinical trials for AD and cerebral amyloid-dependent angiopathy. In this regard, it is worth mentioning that the inactivation of the Nrf2 pathway downregulates downstream antioxidant molecules such as GPX4, further exacerbating not only lipid peroxidation but also iron accumulation and ferroptosis [51]. In fact, the involvement of iron metabolism in the regulation of ferroptosis is associated with molecules considered anti-ferroptotic like Nrf2, which regulates iron metabolism, contributing to the protection from pathological conditions like ischemia-reperfusion, stroke, and other pathologies thus being considered a possible therapeutic target [52].

Indeed, ferroptosis was further defined not only as a chemical process but also associated with the regulation of enzymes including GPX4, the ferroptosis suppressor protein 1 (FSP1) and many others, thus becoming a promising target for specific inducers and inhibitors. FSP1, as a glutathione-independent ferroptosis inhibitor, reduces coenzyme Q10 (ubiquinone) in the mitochondria to the antioxidant ubiquinol (CoQ10H2) which neutralizes free radicals to prevent lipid peroxidation and further suppress ferroptosis.

Modulators of human CAs were also studied in other pathologies, like inflammation, hypoxic tumors, neurodegeneration, and neuropathic pain [53]. A relationship was established between hypoxia, CAs and ischemia [54], and the CAIs of sulfonamide type, with anti-inflammatory properties, exerted protective effects in rats with permanent middle cerebral artery occlusion (pMCAO), already at 24 h after occlusion [54]. Modulation of molecules responsible for acidosis may exert an associated anti-inflammatory effect with influence on neurodegenerative diseases. Cas’ role was shown in the inflammatory response through the regulation of pH through proton generation and the subsequent immune cellular function [55]. The pH homeostasis may thus be modulated by CAIs to reduce H^+^ concentration when the lack of oxygen caused by hypoxia switches glucose metabolism from aerobic to anaerobic, with increased production of lactic acid and a lowered intracellular pH—a mechanism widely studied not only in tumors but also in the ischemic stroke [51,56,57]. As a consequence, the ischemic-hypoxic condition leads to impaired mitochondrial oxidative phosphorylation, with ATP depletion, acidosis and increased electron leakage with ROS production. The latter event is further enhanced by the post-ischemic reperfusion, which activates NF-κB and NLRP3 inflammasome signaling pathways leading to pro-inflammatory cytokine release, activation of ferroptosis, glutathione (GSH) depletion and inhibition of glutathione peroxidase 4 (GPX4) activity [51], further described in Section 4.1 and Section 4.2. Thus, a therapeutic potential may be provided by targeting the ferroptosis pathway to counteract the hypoxic–ischemic injury.

A role of CaA activators has also been suggested in neuropathology, since these enzymes are implicated in the control of pH regulation and cellular respiration. Recent studies support the idea of their involvement in the transmission of neuronal signals [58], thus leading the authors to suggest they may be useful in the treatment of dementia in AD and other cerebral pathologies. Their role in managing diseases is worthy of further investigation.

The pathophysiology of acidosis is related to the regulation of the Bcl-2 family members that aid cells in avoiding apoptosis [59]. Acidosis arises in solid and lymphoid malignancies secondary to altered nutrient supply and utilization. In lymphoma cell lines grown in acidic conditions (pH 6.5), induced apoptosis increases Bcl-2, suggesting that acidosis causes a Bcl-2 dependence, partially mediated by the acid-sensing G protein-coupled receptor GPR65 via a MEK/ERK pathway. In a related mechanism involved during neuronal ischemia, the nuclear factor kappa B (NFκB)/p50/RelA dimer acts as transcriptional inducer of the Bcl-2 proapoptotic family members, while the c-Rel dimers specifically promote the transcription of the anti-apoptotic Bcl-xL gene with changes in the nuclear content of c-Rel dimers. These interactions affect the threshold of neuron vulnerability to ischemic insult and underlie the associated DMT1 contribution to cell death, through epigenetic regulation by NFκB/RelA acetylation at lysine 310 during neuronal and brain ischemia, further described later [60,61,62].

## 3. Acidosis and Iron Uptake by DMT1

Before the identification of DMT1 as an iron importer, characterization of the Belgrade (*b*/*b*) rat and the microcytic (*mk*/*mk*) mouse models led to the conclusion that their mutations severely disrupted metal homeostasis [63,64,65]. Investigation ultimately identified DMT1 as the intestinal importer of iron [1,2]. The *mk* mutation in DMT1 was specified to be G185R, while an identical mutation emerged to account for the *b* phenotype, with both demonstrating a decreased capacity for gastrointestinal and cellular ferrous iron uptake [9]. A diminished expression of DMT1 was observed at the apical membrane of enterocytes in *mk*/*mk* mice [66]. The described animal models with identical mutation G285R in DMT1, which leads to hypochromic microcytic anemia through impaired intestinal iron absorption, were later comforted by the identification of a human mutation of DMT1 in a female with severe hypochromic microcytic anemia [67]. Dissection of the transport of non-Tf iron into cultured cells, performed on both K562 human erythroleukemia cells and a human embryonic kidney cell line (HEK293T), confirmed DMT1-mediated iron uptake via extensive studies of competitive inhibition [68]. After recognition that the transcription of DMT1 mRNA could start with alternative promoters, designated 1A and 1B, four mRNA isoforms were designated: 1A+IRE, 1A-IRE, 1B+IRE and 1B-IRE [17]. These are commonly referred to as alternative splicing isoforms, with protein products that differ at the N- and C-terminal regions with differential tissue expression of the four isoforms finely investigated in mice, where a predominance of 1A/DMT1, both (−)/(+)IRE is shown in the kidney, 1A/(+)IRE DMT1 in the duodenum, and a more diffused expression of 1B/DMT1 in all the other tissues [69], as later summarized.

Of note, the 1A and 1B isoforms were also shown to be differentially expressed not only in yeast, but also in several cell types like the Hep-2, Caco-2 and COS-7, and also in the erythroleukemia cell lines K562 and KU812, although a slightly higher expression of 1B/DMT1 was present, thus implying the regulation of two isoforms is cell type-specific [70]. Interestingly, a deep structural characterization was also initially performed in terms of glycosylation of both 1A and 1B/DMT1 confirming the presence of two predicted N-glycosylation sites, studied by site-directed mutagenesis [70]. Then, the intracellular localization of 1A and 1B/DMT1 was defined by treatment with or without the microtubule-depolymerizing nocodazole, in human larynx carcinoma cell line HEp-2 cells. In this model, 1B/DMT1 stably localizes in the early endosomes, since its colocalization with LAMP2 was abolished by treatment with nocodazole, while 1A/DMT1 was present in the late endosomes and lysosomes [70]. In this regard, the authors also identified an isoform-specific C-terminal domain responsible for lysosomal targeting. In fact, the C-terminal tail of the (−)IRE isoform showed LAMP 2 colocalization in Hep-2 cells, while the (+)IRE isoform was found at the early recycling endosome with TfR colocalization [71]. Studies of ectopic overexpression of different cDNA DMT1 isoforms [17,29] not only characterized some of their properties but also pointed out correctly that they were due to alternative promoters and poly-adenylation sites, with the 1A isoform responsive to hypoxia and downregulated by hepcidin in enterocytes [7] and the 1B isoform transcriptionally upregulated by NFκB and inflammation [60].

Initial evidence revealed that the iron transport activity of DMT1 is pH-dependent with involvement of a H^+^/Fe^2+^ co-transport mechanism and maximal uptake in acidic conditions [1]. Although there has been extensive confirmation of H^+^/Fe^2+^ co-transport [18,72], it is critical to note that it has been argued that proton slippage, a potentially protective mechanism, can occur. While normal H^+^/Fe^2+^ co-transport by DMT1 should have a 1:1 stoichiometry, excess proton concentrations can lead to many protons entering with one ferrous ion [28].

The plasma membrane is not the only sub-cellular location or organelle where acidosis can interact with iron. The mitochondrion can demonstrate expression of all four DMT1 isoforms on the outer mitochondrial membrane (OMM) [16] as shown in mitochondria from the BEAS-2B cell model of normal human bronchial epithelium and in the rat renal cortex. These results provide evidence of the participation of DMT1 in metal uptake (both Fe^2+^ and Mn^2+^) across the OMM [11]. There is also evidence that supports endosomal/mitochondrial contact between the two organelles involved in a pathway for iron uptake in the mitochondria (i.e., the “kiss and run” hypothesis) [12,73,74] (Figure 2). Endosomal acidification is therefore involved in normal iron homeostasis in the cell and organelles, including the mitochondrion [13,75].

Involvement of DMT1 in the accumulation of labile ferrous iron and inflammatory injury is suspected because of its pH-dependent uptake. DMT1 is the elective NTBI importer in mildly acidic environments, as demonstrated in Caco2 cell lines where the uptake of either iron or other divalent metals is maximal at acidic pH values. This function can be selectively abrogated by DMT1 knockdown [23,24,27]. Acidosis-dependent iron uptake is likely to be associated with inflammatory injuries. The mechanism is in relationship to the condition of hypoxia-ischemia, which induces a metabolic shift from aerobic to anaerobic glycolysis, with lactic acid production, lowered intracellular pH, mitochondrial impairment, ROS production, ferroptosis and activation of NF-κB signaling with pro-inflammatory cytokines production [51,56,57]. Accordingly, clear evidence of the acidosis-dependent iron uptake is also provided by experiments done in the condition of experimental acidosis, in differentiated human neuroblastoma cells, where both acute ferrous iron uptake and cell death were increased and the results were further exacerbated after overexpression of 1B/(−)IRE DMT1, even at the basal level in untreated cells [62].

Then, to ascertain the specific mechanistic involvement of the ferrous iron transporter DMT1, the total prevention of cell death was achieved in the differentiated human neuroblastoma model, by (−)IRE DMT1-specific small interfering RNA (siRNA) during in vitro ischemia through oxygen–glucose-deprivation [60,62], a condition producing hypoxic acidosis [51,56,57], also shown to induce the activity of the NF-κB-dependent mouse promoter of the 1B/DMT1 isoform during in vitro ischemia in mouse cortical primary neurons—an induction associated with neuronal cell death [60,62].

Accordingly, control of extracellular and subcellular acidosis is required to avoid increased ferrous iron transport and disruption of metal homeostasis with consequent NTBI overload, activation of ferroptosis and cell death.

**Figure 3:**
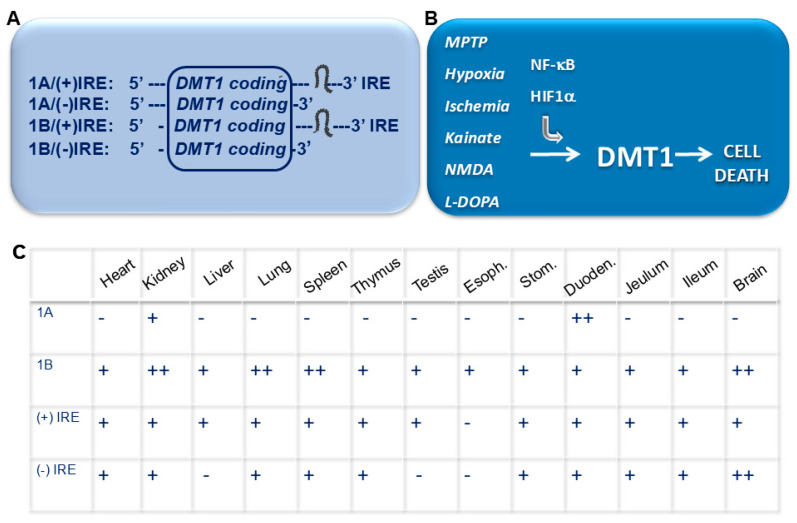
(**A**) 1A and 1B/DMT1 isoforms, with or without the 3′ IRE hairpin stem loop structure, may have different promoters responsive to HIF-1 alpha and NF-κB, respectively, downstream to several excitotoxic stimuli. (**B**,**C**) The 1A and 1B/DMT1 isoforms and the (+)IRE and (−)IRE isoforms, with or without the *Iron Response Element* (IRE) hairpin loop structure, sensitive to intracellular iron levels, are differentially expressed at the mRNA level in different mouse tissues, as previously evaluated.

Indeed, a great effort was spent to define the isoform-specific expression patterns in terms of both tissue, cellular and subcellular distribution which possibly contribute to tissue-specific pathologies. The four isolated isoforms of DMT1 were found differentially expressed, as summarized in Figure 3, through the 1A and 1B alternative promoters, responsive to HIF-1alpha and NFκB, respectively, and downstream to noxious stimuli, as further discussed in Section 4.1 and Section 4.2. The 1A and 1B/DMT1 isoforms, each one with or without the Iron Response Element (IRE) stem loop structure at the 3′ untranslated region, named (−)IRE or (+)IRE, are sensitive to post-translational regulation by intracellular iron levels and differentially expressed at the mRNA level in several rat, mouse and human tissues, as extensively studied [17,62,69,76,77]. The initial evaluation of DMT1 staining by in situ hybridization in the rat brain, performed with a pan-DMT1 probe, identified a heterogenous distribution of the transporter in neurons, mostly in the thalamus, striatum, cerebellum, hippocampal pyramidal and granule layers, anterior olfactory bulb, substantia nigra (pars reticulata and compacta) and choroid plexus in the fourth ventricle, while the lowest signal was found in the cortex [1].

DMT1 expression was also found in the rat brain microvasculature [78,79] and in the monkey hippocampus and neocortex by electron microscopy [80], and a significant neuronal expression was found for (+)IRE DMT1 in the human brain [81]. Furthermore, the analysis of DMT1 with and without IRE led to the identification of a huge expression for both of these isoforms in the mouse kidney, with a predominance of the (−)IRE isoform in the brain [82], in line with later evidence of the predominant 1B/(−)IRE DMT1 isoform, found in the mouse brain and in the human neuroblastoma cell line [62]. Regarding the peripheral expression, DMT1 expression was also localized in the human placenta [83]. A significant difference was then identified for the expression levels of DMT1 with or without IRE, in the Caco2 and Hp3B cell line, with differential regulation by iron treatment or iron chelation [84].

## 4. Acidosis, Iron Homeostasis and Inflammation

### 4.1. Acidosis and Inflammation with Iron Overload

During inflammation, acidosis frequently accompanies an alteration in iron homeostasis with metal accumulation in tissues. A reduction in intracellular pH is associated with inflammatory pathways in macrophages including assembly of the NLR family pyrin domain containing 3 (NLRP3) inflammasome and activation of the chloride intracellular channel protein 1 [85]. Acidosis had been previously implicated in numerous inflammatory processes associated with disruption in iron homeostasis. Hence, one can suggest its role in comorbidities involving this mechanism, perhaps enhancing consequent acidosis-associated injuries in pathological conditions that accompany several neurodegenerative diseases. Extracellular lowering of pH depends on activity of the Na^+^/H^+^-exchanger in cortical neurons, highlighting a possible link between synaptic activity and extracellular pH influencing neuronal activity [45]. DMT1, as a proton co-transporter, has maximal ferrous iron uptake at pH 5.5, elevating iron uptake during pathological extracellular acidosis. This increased flux is key to understanding regulation and dysregulation in acidic conditions. Figure 1 points out the role of acidic conditions in normal iron homeostasis and transition to pathological acidic conditions in certain tissues. Later, however, we will describe how the airways respond to acidosis similarly to neurons by accumulating intracellular iron, though this accumulation has positive downstream effects, at least in the particular models of the Belgrade rat and hypotransferrinemic mouse [76]. Neurons are, nevertheless, prone to negative consequences in all the studies described below. This contrast represents a peculiar aspect of the mechanistic involvement of acidosis, iron overload and inflammation, in relation to physiological responses in different districts, with different implications.

Inflammation stimulates production of the peptide hormone, hepcidin, initially so named because it is secreted by the liver. Now it is understood that many other tissues can generate it for autocrine function. The STAT3/IL-6 and interleukin-1 (IL-1) signaling pathway upregulates its production [86]. This hormone downregulates FPN largely by post-translational degradation, decreasing iron availability in numerous cell types [87,88]. When inflammation occurs in response to infection, iron sequestration is part of the innate immune system. As hepcidin blocks FPN-mediated iron export, iron deficiency downregulates the peptide, allowing compensatory gastrointestinal metal efflux into circulation by enterocytes and a more effective reutilization of effete red blood cells by macrophages. Accordingly, hepcidin controls the concentration of both systemic and tissue metal in its role as an iron-regulatory peptide hormone to avoid consequent cell damage by iron overload and tightly control its homeostasis [7]. In chronic diseases in particular, this process contributes to the anemia of inflammation. In this context, evidence that HAMP directly lowers duodenal DMT1 protein levels [19], confirming earlier reports [20,21], adds new issues that require follow-up data. This new evidence implies that in enterocytes HAMP lowers both iron efflux and influx. If the effect involves it targeting the main intestinal DMT1 isoforms, 1A (−)IRE and 1A (−)IRE, and not the major isoform in most other tissues, then inflammation that increases HAMP in the other tissues will primarily lead to increased iron sequestration. On the other hand, if its effect is pan-isoform-specific for DMT1, then it will lower both influx and efflux in most tissues. The latter possibility seems more unlikely, but an answer to how this hormone affects DMT1 isoforms likely will resolve the question.

Moreover, as HAMP-driven post-translational degradation downregulates FPN levels during inflammation, pharmacological targeting of the BMP/SMAD pathway by heparin derivatives was shown to control HAMP expression in HepG2 cells, in mice after acute lipopolysaccharide (LPS) treatment and in healthy volunteers. Recently, investigators developed a new heparin derivative, sevuparin, with significantly reduced anticoagulant activity [89]. Sevuparin retains HAMP antagonizing activity. It does, however, have residual anticoagulant activity that can lead to secondary events.

Finally, iron overload stimulates the autocatalytic Haber–Weiss reaction and Fenton reaction cycle, releasing the hydroxyl radical and the hydroxide ion, regenerating ferric from ferrous ions. This cycle produces reactive oxygen species (ROS) with oxidative cellular damage to trigger ferroptosis, a distinct form of cell death by iron-dependent lipid peroxidation. Dietary iron overload also decreases oxidative phosphorylation in the liver mitochondria of mice and promotes mitochondrial dysfunction due to oxidative stress, excessive ROS production, consequent lipid peroxidation and cell death. This chain can develop in subjects affected by primary and secondary iron overload, where acidosis and inflammation-dependent iron overload may also be involved in the latter [90]. Indeed, while iron is an essential nutrient, increased amounts make it a potent biohazard by promoting oxidative stress, interfering with physiological signaling pathways associated with tissue injury and disease, particularly in neurodegenerative diseases. It is noteworthy that oxidative stress, which leads to ferroptosis, has a particular influence on neuronal cell types, given the high density of mitochondria [91], where the chemical nature of iron with the electron transport sustains mitochondrial respiration, thus leading to the argument that balanced iron metabolism is imperative for health [92].

### 4.2. Acidosis, Inflammation with Iron Overload and Neurodegeneration

Increasing evidence shows that ferroptosis is involved in aging, infection control, and neurodegenerative diseases with disrupted brain iron homeostasis, such as ischemia-reperfusion injury, neurodegeneration and autoimmune diseases [93]. This point has relevance to acidosis, as pH is tightly regulated at cellular, tissue, and systemic levels; hence, pH in the acidic range comes into play during infection, injury, solid tumors, and physiological and pathological inflammation [94].

Although essential in the function of the CNS, iron is a significant source of ROS, also involved in the neurodegenerative process by damaging neurons with their high content of mitochondria. DMT1 contributes to iron acquisition at many levels of CNS function, and its recently recognized role in mitochondria now has refreshed interest [95]. Interfering with iron entry into mitochondria by disrupting DMT1, which resides on the OMM, renders cells resistant to ferroptosis. This observation has special relevance for neurons, as they are long-lived, nondividing cells. Furthermore, the altered buffering equilibrium favors an increase in the reduced, labile form of iron in acidic conditions. Transport by DMT1, a proton-cotransporter with maximum uptake at pH 5.5, is more effective at such pH values, increasing the intracellular concentration of ferrous ion. Since acidosis is often associated, a decrease in the extracellular pH can support pathological conditions such as inflammation, ischemia, glycolytic cell metabolism or the impaired removal of acidic metabolic byproducts resulting from altered blood perfusion. Downstream effects on the expression of various inflammatory markers and signaling pathways activated in monocytes and macrophages involve mediators, including IL-1ß, IL-6, TNF-α, MCP-1, and COX-2 [96].

Extracellular acidosis is often a hallmark of inflammatory responses to bacterial infection in peripheral tissues, where pH values as low as 5.5 have been detected. Associated tissue hypoxia, the consequent glycolytic switch to lactic acid accumulation, tissue acidification, proton production by neutrophils during the inflammatory response and the generation of short-chain fatty acids synthesized by bacteria all appear to result in this local acidosis [97].

**Figure 4:**
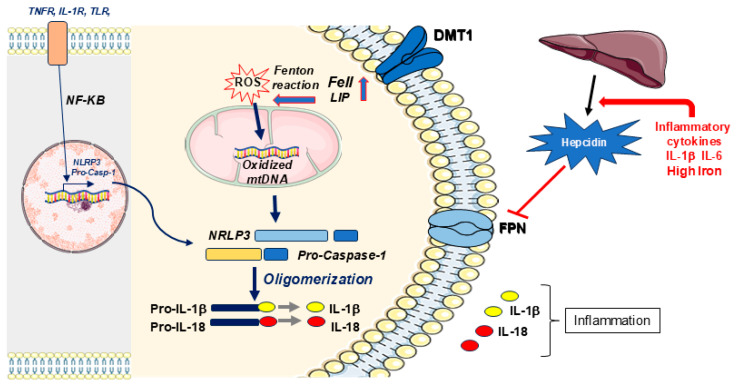
Molecular interactions and signaling pathways involved in the activation of the NLRP3 inflammasome. Interplay with inflammation-dependent activation of iron metabolism. Red arrows: systemic regulation. Black arrows: intracellular signaling.

Acidosis can lead directly to inflammation. Extracellular acidosis activates the NLRP3 (nucleotide-binding domain leucine-rich-containing family, pyrin-domain-containing-3) inflammasome complex. It assembles in response to microbial components or endogenous injury and triggers pH-dependent caspase-1-mediated secretion of IL-1β in human macrophages [98,99,100], together with the release of IL-18, which are strong inducers of inflammation (Figure 4). Moreover, activation of the NLRP3 inflammasome is the basis of many aseptic inflammatory disorders, including diabetes, atherosclerosis, and Alzheimer’s disease. Pro-inflammatory cytokines, IL-1β and IL-6, can induce the secretion of hepcidin, which downregulates FPN expression in monocytes/machrophages, leading to intracellular iron retention. The increased LIP activates the NRLP3 inflammasome and causes long-lasting anemia of chronic inflammation [5,99,101]. Intriguingly, in adipose tissue inflammation, the underestimated impairment of iron homeostasis was recently highlighted, with mitochondrial dysfunction associated with excess iron, particularly ferrous iron with its role in the formation of hydroxyl radicals (OH) via Fenton chemistry, contributing to wide oxidative damage to DNA, lipids and proteins as prodromal factors lead to NLRP3 activation through ROS production and release oxidized mitochondrial DNA—an event at the crossroads between iron dysregulation and cytokine-mediated inflammation with NRLP3 inflammasome activation and oligomerization (Figure 4), downstream to the activation of NF-kB signaling [102]. An NF-kB-dependent mechanism leads to the priming of caspase1, prodromal to the oligomerization and activation of NLRP3 inflammasome. Accordingly, we may recall the NF-kB-dependent 1B/DMT1 isoform [60] with a possible role in the interplay between the iron metabolism and inflammasome activation.

Deliberate alkalinization of extracellular pH significantly inhibited the IL-1β response to NLRP3 activators. In further support of this mechanism, the ginsenoside Rg1 downregulates the expression of inflammatory cytokines induced by LPS and reverses increased expression of Toll-like receptor 4 (TLR4), ROS production, and activation of NFκB, and NOD-like receptor 3 (NLRP3) in cardiac sepsis models in both cardiomyocytes and mice [103]. NFκB and interleukins also contribute, alongside the inflammasome activator NLRP3, to the development of pathological conditions found in neurodegenerative diseases like AD and Parkinson’s disease, where neuroinflammation is recognized as an early hallmark of PD [104]. More recently, a phase 1b open-label study in Parkinson’s patients was conducted with the potent and selective NLRP3 inflammasome inhibitor NT-0796. This isopropyl ester converted to the active metabolite NDT19795 is permeable to the Blood–Brain Barrier, with preclinical efficacy in neuroinflammation [105]. The trial confirmed the anti-neuroinflammatory role of NT-0796 in CNS inhibition of NLRP3, known to be activated by acidosis. It reduced neuroinflammation over a sustained 28-day administration period, thus showing potential for long-term treatment with NT-0796 in future studies of patients with neurodegeneration. NT-0796 was able to reduce the levels of systemic inflammatory markers like C-reactive protein, fibrinogen, interleukin-6 (IL-6), and IL-18 and IL-1β within the CNS of the analyzed subjects with PD [106].

Iron also has exceptional relevance in the inflammatory activation of microglia and infiltrating macrophage. These cells release inflammatory mediators such as cytokines, ROS and hepcidin, all involved in systemic iron regulation [107]. Microglial cells exhibit macrophage-like properties, as part of the CNS innate immune system. During aging, microglial polarization is altered to induce a neurotoxic phenotype with increased expression of pro-inflammatory markers. Thus, dysregulation of microglial–neuronal interaction contributes to sustained microglia activation and neurodegeneration [108]. In this respect, microglia-derived IL-1β during hypoxia and activation of NF-κB signaling pathways were found to be prominent mechanisms at the basis of both glioma progression in nude mice by human glioma xenograft [109], and LPS-induced cognitive impairment with neuroinflammation in C57BL/6J mice [110]. Interleukin-1beta (IL-1β) also contributed after LPS administration in the PD model of 6-hydroxydopamine (6-OHDA)-treated rats, with DMT1 increasing in dopaminergic neurons [111] and in 1-methyl-4-phenylpyridinium (MPP^+^)-treated astrocytes. There, a selective CB_2_ receptor agonist significantly suppressed the MPP^+^-induced upregulation of COX-2, iNOS, IL-1β, and TNF-α and inhibited the MPP^+^-induced upregulation of DMT1 in astrocytes [112]. Like macrophages, microglia contribute either to pro-inflammatory or anti-inflammatory processes in the CNS, reflecting wide-ranging roles in the CNS in maintaining homeostasis and contributing to diseases as well as repair, where altered white matter integrity is associated with microglial dysfunction [113].

Events known to be prodromal to neurodegenerative diseases, like traumatic brain injury, disrupt neurons’ axonal–myelin interactions, causing white matter damage, affecting signal conduction, bioenergetics and plasticity. Such events induce neuroinflammation, possibly leading to the later development of neurodegenerative conditions. Accordingly, microglia, oligodendrocytes and astrocytes—involved in white matter homeostasis—help respond to the traumatic injury [114], with microglial-associated neuroinflammation recognized as an important secondary injury mechanism. This type of damage often persists for many years in humans after initial brain trauma. Similarly, adult mice subjected to single moderate-level cortical impact initially exhibited neuronal loss and microglia activation; when followed up by longitudinal T2-weighted magnetic resonance imaging up to 1 year after traumatic brain injury, they exhibited progressive lesion expansion, hippocampal neurodegeneration, and loss of myelin [115].

After transient middle cerebral artery occlusion, in mouse stroke modes, the presence of the CD11c^+^ marker for microglia in the white matter was associated with spontaneous repair that occurs [116]. Particularly, iron accumulation, implicated in the pathogenesis of demyelination was found in white matter hyperintensities (WMH) analyzed by R2* relaxometry, a technique recently validated in a postmortem study to demonstrate in vivo quantitative brain iron accumulation. WMH volume in MRI and the relaxation rate R2* suggest that iron deposition in the brain reflects the severity [117]. In line with the role of microglial cells responding to inflammation, McCarthy and colleagues [118] provided direct evidence of acidosis increasing the effect of DMT1. They examined murine microglia and a newly immortalized microglial cell line from an adult murine brain to study the response to LPS or Aβ. These pro-inflammatory stimuli led to elevated expression of DMT1 and increased glycolysis, generating an optimal pH for NTBI uptake by the importer. The combined effects caused substantial iron accumulation. In the same model of an immortalized microglial cell line, the investigators also showed that iron exacerbates Aβ-mediated induction of IL-1β through DMT1 expression, since its knockdown by specific siRNA-blocked IL-1β induction [119]. Iron also potentiates the nuclear translocation of NFκB induced by Aβ. Later studies showed a link between pro-inflammatory IL-1, mitochondrial dysfunction and cognitive impairment in AD models, where intravenous injection of Aβ oligomers failed to alter the expression of mitochondrial proteins or memory impairment in Interleukin Receptor-1 knockout (Il1r1−/−) mice [120]. In line with these findings, in vitro DMT1 knockdown has been confirmed to reduce Aβ-dependent inflammatory gene expression and cellular iron levels [121]. The effects of microglial DMT1 (Slc11a2) on AD-related phenotypes in vivo has been elegantly shown in the triple transgenic Cx3cr1^Cre−ERT2^; Slc11a2^flfl^; APP/PS1^+or−^ mice, where the Slc11a2KD APP/PS1 females—but not the males—displayed a significant impairment of memory function in a Morris water maze and in fear conditioning assays, along with hyperactivity compared to control WT and APP/PS1 mice. Hippocampal microglia cells from Slc11a2KD APP/PS1 females also displayed significant increases in FPN gene expression, compared to control APP/PS1 cells, with decreased expression of predominant disease-associated microglia markers such as *Apoe*, *Ctsb*, *Ly9*, *Csf1*, and *Hif1α*, thus defining the gender-specific role of microglial DMT1 in cognitive phenotypes of the AD model of microglial-conditional knockout of DMT1 [121]. The latter intriguing evidence is further supported in BV2 mouse microglial cell lines, where estrogen treatment led to the upregulation of DMT1, FPN and also of the iron storage protein ferritin (FT), together with the upregulation of hypoxia-inducible factor 1 alpha (HIF-1α), while the protein levels of iron regulatory proteins (IRPs) and hepcidin were not affected by the treatment. The latter results are thus support for a Hif-1 alpha-dependent activation of iron metabolism by estrogen signaling and related factors [122]. In this regard, we will later discuss the estrogenic influence on autophagy, involved in neurodegenerative diseases, particularly in the NBIA/BPAN subclass with *de novo* mutations in the WDR45 gene, coding for the beta propeller binding protein (BPAN) responsible for the early phase of the autophagy cascade.

Neuroinflammatory injuries are indeed associated with disrupted brain iron homeostasis, where the principal molecular players for inflammation include NFκB, interleukins, and the inflammasome activator NLRP3, with a downstream regulatory effect on the expression of the NFκB-dependent 1B/DMT1 isoform [60]. These conditions also transcriptionally activate HAMP via IL6, as secretagogue stimulus [123]. HAMP controls the levels of FPN (also expressed in the CNS, where it has been detected in the endothelial cells of the blood–brain barrier, in neurons) in astrocytes, microglial cells, the choroid plexus, ependymal cells—by ultrastructural analysis—presynaptic vesicles of synaptosomal preparations from the rat brain, along the dendrites of Purkinje cells and in the cerebellar molecular layer [124]. Iron dyshomeostasis could thus affect the central nervous system where iron is controlled by physiological conditions with involvement of systemic regulation by HAMP, transferrin-bound iron and NTBI uptake through DMT1, with crosstalk between peripheral districts and the central nervous system [125].

Disrupted iron homeostasis in AD and patients with mild cognitive impairment (MCI) is reflected by altered oxidative stress where iron is a potent source of reactive oxygen species [77] with increased serum ferritin and HAMP. Indeed, both serum polypeptides are also significantly higher among patients with MCI relative to controls, while Aβ40 and Aβ42 decrease [126], showing changes in iron and iron-related proteins associated with the progression of cognitive impairment. These serum biomarkers could potentially contribute to the diagnosis of AD and be indicators of its progression. The correlation supports an interconnection between the CNS and systemic iron homeostasis. More recently, in a murine model of AD [127], the authors found that the initial deposition of Aβ aggregates in the brain is followed by a decline of the Aβ42/Aβ40 ratio in CSF and serum after the cerebral Aβ pathology becomes significant. Their observation is consistent with the existing inverse relationship between HAMP and Aβ levels in the serum among AD and MCI patients.

Deposition of Aβ aggregates, which reflects autophagic impairment, relates to decreased serum levels of ATG5 and Parkin in patients with AD and MCI [128]. Both proteins participate in autophagy and mitophagy with lysosomes acting as acidic organelles. This chain helps to connect disrupted iron homeostasis and the increase in Aβ aggregates to the acidification. The process helps activate autophagy by nutrient starvation prodromal to neurodegeneration, developing after cellular stresses like hypoxia and cytokine inflammation with downstream damage to lysosomes. These results were further supported by deletion of the ATG5 gene in mice, which caused PD-like symptoms [129], implicating the role of autophagy. The mice exhibited impairment in motor coordination and cognitive learning, loss of tyrosine hydroxylase (TH) neurons, and reduction in dopamine levels in the striatum. They also had microglial activation, NLRP3-inflammasome upregulation of downstream IL-1B/IL-1β, and increased expression of the macrophage migration inhibitory factor, a pro-inflammatory cytokine significantly increased in the serum of Parkinson’s patients.

The relationship between PD and an inflammatory injury is also shown in NFκB/c-Rel-deficient mice, a late-onset model of Parkinsonism [130]. Such mice exhibit significant loss of dopaminergic neurons in the substantia nigra pars compacta (SNpc), assessed by disappearance of TH positive fibers, reduced immunoreactivity for the dopamine transporter in the caudate putamen, marked immunoreactivity for fibrillary α-synuclein in the SNpc, increased microglial reactivity in the basal ganglia and age-dependent locomotor deficit [130]. This knockout model of Parkinsonism displayed increased expression of DMT1 and iron staining in both the SNpc and striatum. Consistent observations occur in the PD mouse model of 1-methyl-4-phenyl-1,2,3,6-tetrahydropyridine (MPTP) intoxication, where increased DMT1 expression and concomitant iron accumulation were found in the ventral mesencephalon, with dopaminergic cell loss [131]. Increased expression of DMT1 was also demonstrated in the SNpc of post-mortem slices of PD patients. The *mk* G185R DMT1 mutation in mice provided protection against MPTP intoxication to Parkinsonism; however, in the Belgrade rat model, also carrying the G185R mutation in DMT1 leading to impaired ferrous iron uptake by DMT1, the mutation lessened Parkinsonism induced by the neurotoxin, 6-hydroxydopamine (6-OHDA) [131]. Remarkably, a connection between PD and the post-translational regulation of 1B DMT1, but not 1A DMT1, emerged after the finding that 1B DMT1 was responsive to Parkin-dependent proteasomal degradation [132]. In even earlier studies, Youdim’s lab showed related findings after intranigral iron injections in rats with development of Parkinsonism [133]. This group also implicated excess Fe^3+^, binding neuromelanin with high affinity to potentiate iron-induced lipid peroxidation, by showing that lesion of the nigrostriatal dopamine neurons, induced by 6-OHDA in rats, could be partially prevented by intraventricular injection of the iron chelator deferoxamine [134,135]. Thus, they helped to define the role of iron in PD and the ability of iron chelators to retard dopaminergic neurodegeneration in the SNpc, also proposing moderate iron chelation as a therapeutic approach for PD to avoid great changes in systemic iron levels [136,137]. Careful consideration and tight control of the dynamic equilibrium in the iron homeostasis is relevant to avoid negative influences on both cellular and mitochondrial homeostasis from extreme conditions of either excess iron or its deficiency [138].

Local iron overload also occurs early in the pathogenesis of neurodegenerative diseases mediated by the 1B/(−)IRE isoform of DMT1 after post-ischemic cell death, for example, in cellular and animal models of brain ischemia [61,62]. The relationship between the acidosis-dependent iron overload and the activation of inflammatory signaling through NFκB/RelA was mediated by acetylation at lysine 310, an epigenetic mechanism responsible for post-ischemic cell death in cellular and animal models of brain ischemia. Acidotic post-conditioning in this model is highly toxic for the brain, leading to an inflammatory response, with concomitant iron overload specifically mediated by the NFκB-dependent isoform 1B/(−)IRE DMT1, again with maximum uptake at acidic pH. Thus, acidosis, iron and DMT1 contribute to a complex pattern of interrelationships among aging, neurodegeneration, and neuroinflammation [139].

During ischemia, tissue hypoxia leads to anaerobic glycolysis and lactic acid accumulation, inducing hyperglycemia. Increased lactate usually causes a fall in pH to about 6.0, severely impairing metabolic reoxygenation. Inhibiting lactic acidosis should reduce cellular damage and the related consequences such as mitochondrial malfunction and free radical production [140]. Therefore, inhibiting lactic acidosis may be of therapeutic potential also in respect to the role of ferroptosis during ischemic stroke injury. Such injury can occur due to iron-regulated cell death involving lipid peroxidation, iron accumulation, and downregulation of glutathione peroxidase 4 (GPx4) expression [141]. Increased levels of both ROS and cyclooxygenase 2 (COX2), a key mediator of inflammation, occur during ischemia, linking ferroptosis to cell damage through this pathway, as a possible therapeutic target with contribution of the ferroptosis suppressor protein FSP1FSP1, involved in the myristoylation of the plasma membrane and of several subcellular organelles, which cooperates with the GSH/GPX4 pathway to block iron-regulated cell death and cooperates with the GSH/GPX4 pathway to block iron-regulated cell death. Its involvement during ischemia could make ferroptosis a potential pharmacologically druggable target [141]. Not only the involvement of ferroptosis in in vivo mice models of neurodegenerative diseases has been determined as susceptible to inhibition by GPX4 with rescue of motor neuron survival [142], but, recently, the impairment of ferroptosis and GPX4 function was studied in mitochondrial encephalomyopathy, with lactic acidosis and stroke-like episodes (MELAS) with impaired respiratory function, inhibition of ATP production and increased lactic acid levels due to anaerobic glycolysis during acute attacks [143]. MELAS, which primarily impacts the nervous and muscular systems in patients, shows as a central hallmark of its pathology the imbalance of ferrous iron and mitochondrial damage, the downregulation of the ferroptosis suppressor FSP1, dysregulation of compensatory levels of TfR, decreased DMT1 in fibroblasts of patients, and downregulation of GPX4. The treatment with the iron chelator deferoxamine (DFO) restored cell survival and the mitochondrial fragmentation in MELAS fibroblasts.

Noteworthy in relation to this point, Lorenz et al. elegantly highlighted the significant role of ferroptosis in the development of neurodegenerative diseases [144]. The authors indeed showed the predominant neuroprotective role of GPX4 against ferroptosis and neuroinflammation based on their study of the ultrarare Sedaghatian-type spondylometaphyseal dysplasia (SSMD), which causes autosomal recessive neurodegeneration with skeletal defects due to mutations in the GPX4 gene, with non-complete impact on GPX4 function. This aspect led to development of fine structural studies that highlighted an unrecognized mechanism in GPX4 regulation, well dissected from patient-derived fibroblasts reprogrammed to cerebral cortical neurons and organoids. The fin-loop hydrophobic structure in GPX4 membrane binding was shown to inhibit ferroptosis and its mutation destroys the loop structure and impairs neuroprotection. Then, proteomic analysis of cortical tissues from conditional mouse models clearly linked GPX4 loss of function to neurodegenerative patterns of expression, found not only in Alzheimer disease, but also in Huntington’s (HD), Parkinson’s disease (PD), amyotrophic lateral sclerosis (ALS) and in ferroptosis-related metabolic pathways, which highlights an extended role for ferroptosis in neurodegeneration and neuroinflammation [144]. Furthermore, elevated neuroinflammation occurred in a model of activated astrocytes with excess iron that correlates again with DMT1 function, an increase in iron content and exacerbation of oxidative stress [145]. Also, during aging iron overload can develop, that affects brain function with higher striatal iron associated and diminished fronto-striatal activity in older adults [146]. Inflammation can also cause iron accumulation in the CNS [87], with DMT1 upregulation in the rat hippocampus after excitotoxic injury induced by kainate [147], and the development of Parkinsonism/PD is associated with altered intracellular transport of iron and heavy metals, principally mediated by DMT1 [130,131,139]. This series of observations suggests that NRLP3, activated by extracellular acidosis, triggers the pH-dependent caspase-1-mediated secretion of IL-1 with established influence on DMT1 expression [98]. A further correlation between neuroinflammatory injury and iron is represented by neurodegeneration with brain iron accumulation (NBIA), a heterogeneous group of rare genetic diseases where the principal hallmark is the abnormal and progressive iron accumulation in the basal ganglia [148]. NBIA is classified as a movement disorder with both pediatric and adult onset [149], with twelve disease-related genes presently identified [150,151]. Only two of these genes are directly involved in iron metabolism: FTL (ferritin light chain) and CP (ceruloplasmin) [152,153]; the other ten genes encode proteins with functions either in lipid metabolism, lysosomal activity, autophagic processes, like in the NBIA/BPAN affected patients, with *de novo* mutations in the WDR45 gene coding for the beta-propeller protein-associated neurodegeneration [154] or with still unknown mechanisms. The principal hallmark of NBIA is elevated NTBI accumulation in the basal ganglia detected by MRI [148]. Patients exhibit progressive iron deposition in the SNpc and globus pallidus. Typically, this hallmark initially leads to cerebral atrophy, with bilateral MRI hypointensity emerging later in the globus pallidus and SNpc on T2-weighted sequences with reduced uptake of tracer in DAT-Scan [155,156]. Evidence in support of the influence of extracellular acidosis with iron overload was found in the ischemic penumbra in post-mortem pallidum brain slices from a patient affected by NBIA/PKAN [157], interesting evidence because iron and DMT1 also accumulate in the CNS during post-ischemic events, where significant reduction of cerebral infarct volume in MCAO rats with DMT1 downregualtion are obtained by treatment with Tanshinone IIA, which interferes with the translocation of the active NFκB/p65 subunit from the cytoplasm into the nucleus [158]. In some subclasses of NBIA patients, iron overload was found not only by encephalic MRI analysis, but also in peripheral tissues, like in primary fibroblasts of NBIA/BPAN-affected patients with rare X-linked *de novo* mutations in the WD repeat domain 45 (WDR45) gene, encoding for the beta-propeller binding protein WIPI4, one of the mammalian homologs of yeast Atg18. WIPI4 has an important role in autophagy, as demonstrated in lymphoblastoid cell lines of the affected subjects, thus defining the impairment of autophagy in NBIA/BPAN as the principal hallmark of the pathology [159]. Indeed, primary fibroblasts of NBIA/BPAN patients exhibit impaired intracellular iron homeostasis with an altered pattern of iron transporters, downregulation of Transferrin Receptor 1 and upregulation of endogenous (−)IRE/DMT1. An increase in ferrous iron was shown by Turnbull’s staining in cells exposed to starvation [160], a condition that not only reactivates autophagy, but also induces acidosis, mimicking a metabolic state associated with high levels of ketones as energy resources in the blood in long-lasting carbohydrate deficiency [161]. The results were later confirmed in a WDR45-knockout model produced in the SH-SY5Y neuroblastoma cell line by CRISPR-Cas9-mediated genome editing [162], where cells and their mitochondria accumulated iron after upregulation of (−)IRE-DMT1 mRNA. These changes led to impaired mitochondrial respiration, increased ROS, cell death and diminished extracellular acidification rate, with reduced basal glycolysis and glycolytic capacity. This model of WDR45 deficiency promoted general impairment in cellular metabolism without a compensatory mechanism leading to the expected metabolic shift to glycolysis [163], and was further confirmed by WDR45 siRNA in both HeLa and SH-SY-5Y neuroblastoma cells which induced loss of function of the WDR45 gene and increased cell death, measured by lactate dehydrogenase (LDH) release in SH-SY5Y neuroblastoma, rescued by ferroptosis inhibitors [164].

**Figure 5:**
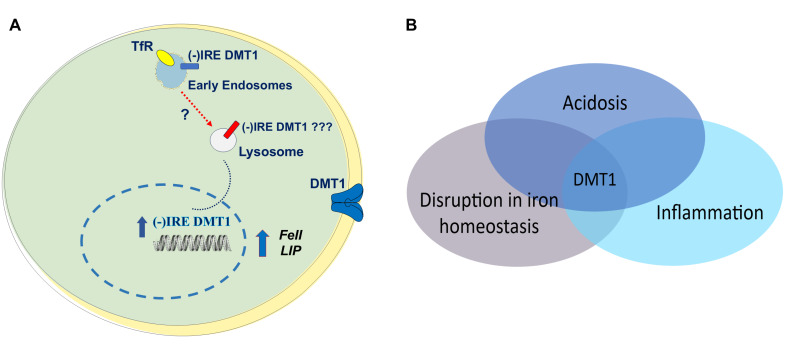
(**A**) Upregulation of (−)IRE DMT1, known to be colocalized with early recycling endosomes, and its hypothetic intracellular derangement to LAMP2-positive lysosomal structures. (**B**) A Venn-like diagram of the impact between acidic conditions, disruption of iron homeostasis and inflammation.

Hence, we may also infer that extracellular acidification after cellular stressors, like hypoxia, nutrient deprivation, and cytokine inflammation, impacts lysosomal and autophagic functions also in non-cancerous cells [165]. In fact, disturbed iron metabolism correlates with inflammation and lysosomal impairment, consequent to autophagic derangement, as widely described (Figure 5B).

Future experiments will possibly establish a more defined mechanism of acidosis with ferrous iron and (−)IRE DMT1 involvement, being a specific isoform upregulation, with possible derangement of its intracellular trafficking from the TfR-positive early endosomes to the LAMP2-positive lysosomal structures, when associated with autophagy impairment like in NBIA/BPAN neurodegeneration or more, as hypothesized (Figure 5A).

Moreover, the WDR45-dependent impairment of the cell cycle was also shown in primary fibroblasts of BPAN patients [166], associated with downregulation of Cyclin B1, increased Caveolin 1 and reduced protein levels of the transcription factor EB (TFEB), the master regulator of autophagy, whose silencing was found to block the G1/S checkpoint of the cell cycle in non-cancerous-cells [167]. TFEB overexpression in the fibroblasts of BPAN patients indeed rescued the impairment in growth rate identified, possibly implying a related influence in the central component of glial cells. While little is known about the relationship between cell cycle and autophagy, we may asses that in the WDR45 model, that shows autophagy impairment as the principal hallmark of NBIA/BPAN, TFEB downregulation is involved. TFEB overexpression in PC12 cell line was indeed shown to counteract the increase in ferrous iron uptake and intracellular LIP, preventing ferroptosis [168], thus confirming the contribution of TFEB in the control of intracellular iron.

Not least, of interest is the consideration that NBIA/BPAN-affected subjects are mostly females, the pathology determined by X-linked mutations, with upregulation of intracellular iron and DMT1, impaired autophagy and TFEB downregulation. We may thus argue a possible gender-related hypothesis, not only due to the factors involved in iron metabolism under the control of estrogens and hypoxia, as already mentioned [121], but also in light of the more recent finding showing TFEB as a direct target gene of the estrogen receptor-related orphan receptors (ERRs) and induced by ERR agonists in both neonatal rat ventricular myocytes and C_2_C_12_ myoblasts [169]. TFEB, as an estrogen target, could thus represent a promising targeting strategy to overcome the impaired mechanism showed not only in the NBIA/BPAN models but possibly also in the other neurodegenerative diseases with impairment of autophagy, associated with a gender-related phenotype.

The picture that emerges for this section is that inflammatory challenges frequently involve acidosis leading to increased iron sequestration in neurological tissues. The process leads to neurodegeneration and diminishing this damage becomes an obvious goal.

If other tissues maintained this paradigm, management of inflammatory local iron accumulation would be relatively straightforward. The next section shows that iron researchers face a more complicated challenge.

### 4.3. Acidosis and Inflammation with Iron Overload in Airways

Disorders involving low pH in exhaled breath condensate (EBC) in patients with inflammatory pulmonary disease and disruption in iron homeostasis include asthma, chronic obstructive pulmonary disease, interstitial lung disease, cystic fibrosis, and lung transplantation [170,171,172,173,174,175,176,177,178,179,180,181]. Acidosis in these inflammatory lung diseases can be normalized with treatment [182]. A low pH in EBC correlates well with indices of airway inflammation including eosinophilia and neutrophilia [183]. Smoking, similarly, leads to acidosis, disruption in iron homeostasis, and inflammatory injury in the environment of the lower respiratory tract [184,185,186,187].

Iron import into both epithelial cells and macrophages in the lower respiratory tract can depend on DMT1; this importer also plays an important role in pulmonary inflammatory responses [188,189,190]. The expression of DMT1 in the lung increases with inflammatory injury [191]. The coordinated handling of iron appears to diminish the potential for oxidative stress, and therefore inflammatory injury, associated with the metal [76].

Metabolic acidosis in systemic inflammatory injuries can include lactic acidosis [192], which may not solely reflect hypoxia in the lung [193]. Under conditions of altered iron availability, cells and tissues evolved mechanisms to acquire iron. A deficiency of metal stimulates the reprogramming of carbon metabolism, increasing the activity of enzymes involved in the Krebs cycle and the glycolytic pathway. The resultant carboxylates/hydroxycarboxylates then function as ligands to complex iron and facilitate solubilization and uptake, reversing absolute or functional metal deficiency. Cells produce lactate, a hydroxycarboxylate, to import metal [194]. Consequently, lactic acidosis alters iron homeostasis in inflammatory injuries of the airways [195,196]. Hyperlactatemia and anemia commonly coexist, further supporting the relationship between acidosis and iron homeostasis [197]. Acidosis other than lactic acidosis may also be associated, however, with disruptions in iron homeostasis and inflammatory injuries [198].

Altering the expression or function of proton pumps and pH can impact inflammatory injury in tissues and these same enzymes also affect iron homeostasis [199]. The H^+^-ATPases are involved in numerous inflammatory injuries associated with disrupted iron homeostasis, including asthma and infections, as well as cancer [200,201,202,203,204,205].

Hence, acidosis is frequently accompanied by iron accumulation in tissues other than the duodenum, usually under inflammatory or stressful circumstances. That accumulation in the airways apparently has a protective effect. There needs to be further investigation to ascertain the extent to which the pairing is a metabolic effort to compensate or whether it is itself pathological.

## 5. Iron Chelation and Transport Strategies

Treatments can target iron accumulation, DMT1 activity or acidification. Targeting DMT1 introduces an important problem. The G185R mutation in DMT1 protects against models of Parkinsonism [131], supporting the concept that in the CNS (SNpc, in particular) it contributes to the pathology of PD. In contrast, however, the same mutation in DMT1 leads to increased inflammatory injury in the airways [76], consistent with intracellular iron accumulation playing a protective role in this arena. Strategies involving treatment are complicated by this contrast, and more research needs to be done to understand the differences and learn what causes the contrast as well as how other tissues and disease states respond. Perhaps this difference could relate to the recent finding mentioned twice above that hepcidin directly regulates duodenal DMT1 where the responding protein isoform is 1A (+IRE). We need to know first whether hepcidin similarly affects some other isoforms and whether distinct isoforms occur in airways versus neural tissues. As acidosis certainly has other consequences (and the increased effectiveness of DMT1 can also lead to metals other than iron accumulating when they are available to the transporter), our concept definitely merits further investigation. Targeted interventions can involve enhancing, removing or blocking iron accumulation depending on which paradigm has support.

Burke et al. proposed using small molecules to replace mutated gene products like transporters or enzymes when concentration gradients could still drive the altered process [206]. Among these, the natural product hinokitiol can increase iron transport in specific animal models with a mutated iron transporter. Subsequently, when compared to different isoforms of DMT1 in HEK293 cell lines capable of overexpressing them, hinokitiol was found to transport both Fe^2+^ and Fe^3+^ with Michaelis–Menten kinetics [207].

Initial reports described ebselen as an organo-selenium compound with GPx-like, thiol-dependent, hydroperoxide-reducing activity [208,209]. Subsequently, in clinical trials, this seleno-compound was found to be protective against ischemic stroke [210,211]. Later work identified ebselen as a small-molecule inhibitor of iron uptake via DMT1 [212]. Inhibition of Fe(II) uptake by ebselen, with an IC(50) ~0.22 µM, did not influence Fe(III) transport or DMT1-mediated manganese uptake. Ebselen was also effective against peripheral oxidative stress in an animal model of sporadic AD induced by intracerebroventricular streptozotocin administration [213]. More recently, ebselen was found to protect against iron-overload-associated cardiomyopathy in thalassemic patients by lowering the iron burden [214]. In a model of senescence induced by ferric ammonium citrate treatment in human SH-SY5Y neuroblastoma cells, ebselen was found to influence the levels of DMT1, ferritin light chains, and cytosolic iron, while increasing FPN expression [215].

DMT1 knockdown was found to abrogate ferrous iron uptake selectively in Caco2 cell lines [17,27], suggesting another option for DMT1 management. In a mouse model of hemochromatosis, gavage of ginger nanoparticles targeting the duodenum successfully delivered DMT1-specific siRNA to lower intestinal expression levels [216,217]. This treatment mitigated iron overload without apparent toxicity. In another investigation, siRNA to (−)IRE DMT1 protected against cell death during the experimental model of ischemia, like oxygen–glucose-deprivation (OGD) in differentiated human SK-NS-H neuroblastoma cells [62]. Inhibition of iron overload was evaluated using total X-ray fluorescence analysis. In the same model, neuroprotection was also obtained by iron chelation with desferal during OGD and post-ischemic reoxygenation [62]. Animal models with G185R mutation in DMT1 demonstrated resistance to induced Parkinsonism [131]. These and other data led to trials utilizing deferiprone chelation in PD patients shortly after diagnosis [218]. Although neuroimaging evaluation detected a greater decrease in nigrostriatal iron content in the deferiprone group than in the placebo group, treatment led to a worsening of motor and nonmotor symptoms during 36 weeks of observation. A multifactorial neurodegenerative disorder like PD may require a combination of treatments to target different mechanistic pathways simultaneously [138]. These authors also inferred that the trial may have overshot on deferiprone chelation. A recent review [219] highlighted brain iron elevations in patients with PD, AD, NBIA and ALS. The failures of deferiprone chelation including those cited above and by them allow us to point out that the view of iron as a double-edged sword due to pathology when deficient or elevated leaves health balanced on the sword edge. They review how iron dyshomeostasis can interact with needed brain functions in the disorders and begin to deal with how these interactions can be managed. Plausibly, this pair of analogies—a double-edged sword and balancing on the sword edge—is behind the paradoxical difference between lung [76] versus brain. In the sex-specific phenotype of cognitive impairment by iron deprivation in the microglial conditional knockout of DMT1 [121], estrogen appears decisive for pushing to one side [121].

A subsequent trial treating Alzheimer’s dementia with deferiprone chelation, under the same conditions as the previously described PD trial, noted comparable results regarding worsening in the chelation group [220]. It is possible that hinokitiol [207] may be easier to control than deferiprone, allowing optimal levels of iron, in consideration of the tight control of iron homeostasis [138].

With several CaA isoforms expressed in the brain, specific inhibitors that modulate acidosis represent possible pharmacological compounds for protection during brain ischemia [221,222]. Other recent approaches are currently under evaluation such as small-molecule inhibitors of the NRLP3 inflammasome also targeting the NLRP3 nucleotide-binding and oligomerization domain to improve solubility and clinical efficacy [223,224]. Another inhibitor approach to managing neurodegeneration could be to take advantage of the role of degradation by Parkin and other E3 ligases, which is reported to affect ferroptosis [225]. Such an approach is partly inspired by the investigation in [132] in which Parkin overexpression in human neuroblastoma was found to reduce 1B-DMT1, associated with divalent metal transport and toxicity. The proteasomal inhibitor MG-132 counteracted all these effects.

## 6. Conclusions and Notice

A mildly acidic cellular environment supports maximal uptake of iron. This observation is true for intestinal enterocytes as well as for the cells of other tissues. Inflammation of other tissues is often associated with acidosis, potentially affecting the homeostasis of this metal and increasing intracellular iron uptake. While such a response can be normal for enterocytes, it can often be overwhelming for the cells of other tissues leading to both beneficial and harmful roles (Figure 5B), thus suggesting a more coordinated evaluation is needed between iron uptake, metabolic acidosis and inflammation as an upstream driver of the explored mechanisms

We made a preprint [226] available with a description of the concept that acid conditions can accelerate iron transport to encourage studies based on the concept. The current review clearly differs substantially.

## Data Availability

No new data were created or analyzed in this study. Data sharing is not applicable to this article.

## Abbreviations

The following abbreviations are used in this manuscript:

DMT1	divalent metal transporter 1
NTBI	non-transferrin bound iron
NBIA	neurodegeneration with brain iron accumulation
pMCAO	permanent middle cerebral artery occlusion
BPAN	beta propeller binding protein
IRE	iron-responsive element
ATP4	H(+)/K(+) ATPase
ASICs	acid-sensing ion channels
CaAs	carbonic anhydrases
CAIs	carbonic anhydrase inhibitors
FPN	ferroportin
Tf	transferrin
TfR	transferrin receptor
FtL	ferritin light chain
HAMP	hepcidin
Hp	hephaestin
Cp	ceruloplasmin
Nrf2	nuclear factor erythroid 2-related factor 2
OMM	outer mitochondrial membrane
LIP	labile iron pool
NLRP3	NLR family pyrin domain containing 3
TLR4	toll-like receptor 4
GPx4	glutathione peroxidase 4
SNpc	substantia nigra pars compacta
HIF-1α	hypoxia inducible factor 1 alpha
EBC	exhaled breath condensate

## References

[1] Gunshin H., Mackenzie B., Berger U.V., Gunshin Y., Romero M.F., Boron W.F., Nussberger S., Gollan J.L., Hediger M.A. (1997). Cloning and characterization of a mammalian proton-coupled metal-ion transporter. Nature.

[2] Fleming M.D., Trenor C.C., Su M.A., Foernzler D., Beier D.R., Dietrich W.F., Andrews N.C. (1997). Microcytic anaemia mice have a mutation in Nramp2, a candidate iron transporter gene. Nat. Genet..

[3] Philpott C.C., Ryu M.S. (2014). Special delivery: Distributing iron in the cytosol of mammalian cells. Front. Pharmacol..

[4] Yanatori I., Kishi F. (2019). DMT1 and iron transport. Free Radic. Biol. Med..

[5] Ganz T. (2013). Systemic iron homeostasis. Physiol. Rev..

[6] Nemeth E., Tuttle M.S., Powelson J., Vaughn M.B., Donovan A., McVey Ward D., Ganz T., Kaplan J. (2004). Hepcidin regulates cellular iron efflux by binding to ferroportin and inducing its internalization. Science.

[7] Nemeth E., Ganz T. (2021). Hepcidin-Ferroportin interaction controls systemic iron homeostasis. Int. J. Mol. Sci..

[8] Knutson M.D. (2017). Iron transport proteins: Gateways of cellular and systemic iron homeostasis. J. Biol. Chem..

[9] Fleming M.D., Romano M.A., Su M.A., Garrick L.M., Garrick M.D., Andrews N.C. (1998). Nramp2 is mutated in the anemic Belgrade (b) rat: Evidence of a role for Nramp2 in endosomal iron transport. Proc. Natl. Acad. Sci. USA.

[10] Garrick L., Romano M., Watkins A., Glass J., Garrick M. (1997). Endosomes Isolated from Belgrade Rats exhibit multiple defects. Blood.

[11] Wolff N.A., Garrick M.D., Zhao L., Garrick L.M., Ghio A.J., Thévenod F. (2018). A role for divalent metal transporter (DMT1) in mitochondrial uptake of iron and manganese. Sci. Rep..

[12] Dhungana S., Taboy C.H., Zak O., Larvie M., Crumbliss A.L., Aisen P. (2004). Redox properties of human transferrin bound to its receptor. Biochemistry.

[13] Barra J., Crosbourne I., Roberge C.L., Bossardi-Ramos R., Warren J.S.A., Matteson K., Wang L., Jourd’heuil F., Borisov S.M., Bresnahan E. (2024). DMT1-dependent endosome-mitochondria interactions regulate mitochondrial iron translocation and metastatic outgrowth. Oncogene.

[14] Hentze M.W., Kühn L.C. (1996). Molecular control of vertebrate iron metabolism: mRNA-based regulatory circuits operated by iron, nitric oxide, and oxidative stress. Proc. Natl. Acad. Sci. USA.

[15] Pantopoulos K. (2004). Iron metabolism and the IRE/IRP regulatory system: An update. Ann. N. Y. Acad. Sci..

[16] Wolff N.A., Ghio A.J., Garrick L.M., Garrick M.D., Zhao L., Fenton R.A., Thévenod F. (2014). Evidence for mitochondrial localization of divalent metal transporter 1 (DMT1). FASEB J..

[17] Garrick M.D., Dolan K.G., Horbinski C., Ghio A.J., Higgins D., Porubcin M., Moore E.G., Hainsworth L.N., Umbreit J.N., Conrad M.E. (2003). DMT1: A mammalian transporter for multiple metals. Biometals.

[18] Mackenzie B., Garrick M.D. (2005). Iron imports. II. Iron uptake at the apical membrane in the intestine. Am. J. Physiol. Gastrointest. Liver Physiol..

[19] Falabrègue M., Aurrand C., Cazaulon L., Boussetta N., Zumerle S., Karim Z., Djebrani-Oussedik N., Poupon J., Guilmeau S., Dupe E. (2025). Intestinal hepcidin overexpression promotes iron deficiency anemia and counteracts iron overload via DMT1 downregulation. Blood.

[20] Mena N.P., Esparza A., Tapia V., Valdés P., Núñez M.T. (2008). Hepcidin inhibits apical 276 iron uptake in intestinal cells. Am. J. Physiol. Gastrointest. Liver Physiol..

[21] Brasse-Lagnel C., Karim Z., Letteron P., Bekri S., Bado A., Beaumont C. (2011). Intestinal DMT1 cotransporter is down-regulated by hepcidin via 274 proteasome internalization and degradation. Gastroenterology.

[22] Collins J.F. (2025). Luminal hepcidin targets intestinal DMT1. Blood.

[23] Garrick M.D., Dolan K.G., Armstrong D. (2002). An expression system for a transporter of iron and other metals. Ultrastructure and Molecular Biology for Oxidants and Antioxidants; Methods in Molecular Biology.

[24] Garrick M.D., Kuo H.C., Vargas F., Singleton S., Zhao L., Smith J.J., Paradkar P., Roth J.A., Garrick L.M. (2006). Comparison of mammalian cell lines expressing distinct isoforms of divalent metal transporter 1 in a tetracycline-regulated fashion. Biochem. J..

[25] Arredondo M., Mendiburo M.J., Flores S., Singleton S.T., Garrick M.D. (2014). Mouse divalent metal transporter 1 is a copper transporter in HEK293 cells. Biometals.

[26] Garrick M.D., Singleton S.T., Vargas F., Kuo H.C., Zhao L., Knöpfel M., Davidson T., Costa M., Paradkar P., Roth J.A. (2006). DMT1: Which metals does it transport?. Biol. Res..

[27] Bannon D.I., Abounader R., Lees P.S., Bressler J.P. (2003). Effect of DMT1 knockdown on iron, cadmium, and lead uptake in Caco-2 cells. Am. J. Physiol. Cell Physiol..

[28] Nevo Y., Nelson N. (2006). The NRAMP family of metal-ion transporters. Biochim. Biophys. Acta.

[29] McKie A.T., Barrow D., Latunde-Dada G.O., Rolfs A., Sager G., Mudaly E., Mudaly M., Richardson C., Barlow D., Bomford A. (2001). An iron-regulated ferric reductase associated with the absorption of dietary iron. Science.

[30] Daher R., Ducrot N., Lefebvre T., Zineeddine S., Ausseil J., Puy H., Karim Z. (2022). Crosstalk between Acidosis and Iron Metabolism: Data from In Vivo Studies. Metabolites.

[31] Schwarz P., Kübler J.A., Strnad P., Müller K., Barth T.F., Gerloff A., Feick P., Peyssonnaux C., Vaulont S., Adler G. (2012). Hepcidin is localized in gastric parietal cells, regulates acid secretion and is induced by *Helicobacter pylori* infection. Gut.

[32] Hamano H., Niimura T., Horinouchi Y., Zamami Y., Takechi K., Goda M., Imanishi M., Chuma M., Izawa-Ishizawa Y., Miyamoto L. (2020). Proton pump inhibitors block iron absorption through direct regulation of hepcidin via the aryl hydrocarbon receptor-mediated pathway. Toxicol. Lett..

[33] Khatib M.A., Rahim O., Kania R., Molloy P. (2021). Iron deficiency anemia induced by long-term ingestion of omeprazole. Dig. Dis. Sci..

[34] Lam J.R., Schneider J.L., Quesenberry C.P., Corley D.A. (2017). Proton pump inhibitor and histamine-2 receptor antagonist use and Iron deficiency. Gastroenterology.

[35] Lampe R.H., Coale T.H., Forsch K.O., Jabre L.J., Kekuewa S., Bertrand E.M., Horák A., Oborník M., Rabines A.J., Rowland E. (2023). Short-term acidification promotes diverse iron acquisition and conservation mechanisms in upwelling-associated phytoplankton. Nat. Commun..

[36] Nuñez M.T., Gaete V., Watkins J.A., Glass J. (1990). Mobilization of iron from endocytic vesicles. The effects of acidification and reduction. J. Biol. Chem..

[37] Watkins J.A., Nunez M.T., Gaete V., Alvarez O., Glass J. (1991). Kinetics of iron passage through subcellular compartments of rabbit reticulocytes. J. Membr. Biol..

[38] Diab H.I., Kane P.M. (2013). Loss of vacuolar H+-ATPase (V-ATPase) activity in yeast generates an iron deprivation signal that is moderated by induction of the peroxiredoxin TSA2. J. Biol. Chem..

[39] Ohgami R.S., Campagna D.R., Greer E.L., Antiochos B., McDonald A., Chen J., Sharp J.J., Fujiwara Y., Barker J.E., Fleming M.D. (2005). Identification of a ferrireductase required for efficient transferrin-dependent iron uptake in erythroid cells. Nat. Genet..

[40] Sendamarai A.K., Ohgami R.S., Fleming M.D., Lawrence C.M. (2008). Structure of the membrane proximal oxidoreductase domain of human Steap3, the dominant ferrireductase of the erythroid transferrin cycle. Proc. Natl. Acad. Sci. USA.

[41] Forgac M. (2007). Vacuolar ATPases: Rotary proton pumps in physiology and pathophysiology. Nat. Rev. Mol. Cell Biol..

[42] Cheng Y.R., Jiang B.Y., Chen C.C. (2018). Acid-sensing ion channels: Dual function proteins for chemo-sensing and mechano-sensing. J. Biomed. Sci..

[43] Mazzocchi N., De Ceglia R., Mazza D., Forti L., Muzio L., Menegon A. (2016). Fluorescence-Based automated screening assay for the study of the pH-sensitive channel ASIC1a. J. Biomol. Screen..

[44] Mango D., Nisticò R. (2021). Neurodegenerative disease: What potential therapeutic role of acid-sensing ion channels?. Front. Cell. Neurosci..

[45] Chiacchiaretta M., Latifi S., Bramini M., Fadda M., Fassio A., Benfenati F., Cesca F.J. (2017). Neuronal hyperactivity causes Na^+^/H^+^ exchanger-induced extracellular acidification at active synapses. J. Cell Sci..

[46] Xiong Z.G., Xu T.L. (2012). The role of ASICS in cerebral ischemia. Wiley Interdiscip. Rev. Membr. Transp. Signal..

[47] López-Ramírez O., González-Garrido A. (2023). The role of acid sensing ion channels in the cardiovascular function. Front. Physiol..

[48] Redd M.A., Scheuer S.E., Saez N.J., Yoshikawa Y., Chiu H.S., Gao L., Hicks M., Villanueva J.E., Joshi Y., Chow C.Y. (2021). Therapeutic inhibition of acid-sensing ion channel 1a recovers heart function after ischemia-reperfusion injury. Circulation.

[49] Lemon N., Canepa E., Ilies M.A., Fossati S. (2021). Carbonic anhydrases as potential targets against neurovascular unit dysfunction in alzheimer’s disease and stroke. Front. Aging Neurosci..

[50] Canepa E., Parodi-Rullan R., Vazquez-Torres R., Gamallo-Lana B., Guzman-Hernandez R., Lemon N.L., Angiulli F., Debure L., Ilies M.A., Østergaard L. (2023). FDA-approved carbonic anhydrase inhibitors reduce amyloid β pathology and improve cognition, by ameliorating cerebrovascular health and glial fitness. Alzheimer’s Dement..

[51] Qi L., Yi J., Shen Y., Jiang H., Yao X., Chen B., Sun H. (2026). The myocardial ischemic cascade network and multi-target synergistic interventions: From molecular mechanisms to therapeutic innovations. Biochem. Pharmacol..

[52] Berndt C., Alborzinia H., Amen V.S., Ayton S., Barayeu U., Bartelt A., Bayir H., Bebber C.M., Birsoy K., Böttcher J.P. (2024). Ferroptosis in health and disease. Redox Biol..

[53] Giovannuzzi S., Supuran C.T. (2025). Human carbonic anhydrase modulators: The past, present, and future. Trends Pharmacol. Sci..

[54] Mannelli L.D.C., Micheli L., Carta F., Cozzi A., Ghelardini C., Supuran C.T. (2016). Carbonic anhydrase inhibition for the management of cerebral ischemia: In Vivo evaluation of sulfonamide and coumarin inhibitors. J. Enzym. Inhib. Med. Chem..

[55] Cao L., Huang T., Chen X., Li W., Yang X., Zhang W., Li M., Gao R. (2021). Uncovering the interplay between pH receptors and immune cells: Potential drug targets. Oncol. Rep..

[56] Swietach P., Vaughan-Jones R.D., Harris A.L. (2007). Regulation of tumor pH and the role of carbonic anhydrase 9. Cancer Metastasis Rev..

[57] Wang Y., Ge M., Wang J., Xu Y., Wang N., Xu S. (2025). Metabolic reprogramming in ischemic stroke: When glycolytic overdrive meets lipid storm. Cell Death Dis..

[58] Poggetti V., Salerno S., Baglini E., Barresi E., Da Settimo F., Taliani S. (2022). Carbonic anhydrase activators for neurodegeneration: An overview. Molecules.

[59] Ryder C., McColl K., Zhong F., Distelhorst C.W. (2012). Acidosis promotes Bcl-2 family-mediated evasion of apoptosis: Involvement of acid-sensing G protein-coupled receptor Gpr65 signaling to Mek/Erk. J. Biol. Chem..

[60] Paradkar P.N., Roth J.A. (2006). Nitric oxide transcriptionally down-regulates specific isoforms of divalent metal transporter (DMT1) via NF-kappaB. J. Neurochem..

[61] Sarnico I., Lanzillotta A., Benarese M., Alghis M., Baiguera C., Battistin L., Spano P.F., Pizzi M. (2009). NF-kappaB dimers in the regulation of neuronal survival. Int. Rev. Neurobiol..

[62] Ingrassia R., Lanzillotta A., Sarnico I., Benarese M., Blasi F., Borgese L., Bilo F., Depero L., Chiarugi A., Spano P.F. (2012). 1B/(−)IRE DMT1 expression during brain ischemia contributes to cell death mediated by NF-κB/RelA acetylation at Lys310. PLoS ONE.

[63] Oates P.S., Morgan E.H. (1996). Defective iron uptake by the duodenum of Belgrade rats fed diets of different iron contents. Am. J. Physiol. Gastrointest. Liver Physiol..

[64] Garrick M., Scott D., Walpole S., Finkelstein E., Whitbred J., Chopra S., Trivikram L., Mayes D., Rhodes D., Cabbagestalk K. (1997). Iron supplementation moderates but does not cure the Belgrade anemia. BioMetals.

[65] Edwards J.A., Hoke J.E. (1972). Defect of intestinal mucosal iron uptake in mice with hereditary microcytic anemia. Proc. Soc. Exp. Biol. Med..

[66] Canonne-Hergaux F., Fleming M.D., Levy J.E., Gauthier S., Ralph T., Picard V., Andrews N.C., Gros P. (2000). The Nramp2/DMT1 iron transporter is induced in the duodenum of microcytic anemia mk mice but is not properly targeted to the intestinal brush border. Blood.

[67] Mims M.P., Guan Y., Pospisilova D., Priwitzerova M., Indrak K., Ponka P., Divoky V., Prchal J.T. (2005). Identification of a human mutation of DMT1 in a patient with microcytic anemia and iron overload. Blood.

[68] Conrad M.E., Umbreit J.N., Moore E.G., Hainsworth L.N., Porubcin M., Simovich M.J., Nakada M.T., Dolan K., Garrick M.D. (2000). Separate pathways for cellular uptake of ferric and ferrous iron. Am. J. Physiol. Gastrointest. Liver Physiol..

[69] Hubert N., Hentze M.W. (2002). Previously uncharacterized isoforms of divalent metal transporter (DMT)-1: Implications for regulation and cellular function. Proc. Natl. Acad. Sci. USA.

[70] Tabuchi M., Tanaka N., Nishida-Kitayama J., Ohno H., Kishi F. (2002). Alternative splicing regulates the subcellular localization of divalent metal transporter 1 isoforms. Mol. Biol. Cell.

[71] Tabuchi M., Yanatori I., Kawai Y., Kishi F. (2010). Retromer-mediated direct sorting is required for proper endosomal recycling of the mammalian iron transporter DMT1. J. Cell Sci..

[72] Shawki A., Anthony S.R., Nose Y., Engevik M.A., Niespodzany E.J., Barrientos T., Öhrvik H., Worrell R.T., Thiele D.J., Mackenzie B. (2015). Intestinal DMT1 is critical for iron absorption in the mouse but is not required for the absorption of copper or manganese. Am. J. Physiol. Gastrointest. Liver Physiol..

[73] Das A., Nag S., Mason A.B., Barroso M.M. (2016). Endosome-mitochondria interactions are modulated by iron release from transferrin. J. Cell Biol..

[74] Sheftel A.D., Zhang A.S., Brown C., Shirihai O.S., Ponka P. (2007). Direct interorganellar transfer of iron from endosome to mitochondrion. Blood.

[75] Garrick M.D., Gniecko K., Liu Y., Cohan D.S., Garrick L.M. (1993). Transferrin and the transferrin cycle in Belgrade rat reticulocytes. J. Biol. Chem..

[76] Ghio A.J., Wang X., Dailey L.A., Stonehuerner J.D., Piantadosi C.A., Yang F., Dolan K.G., Garrick L.M., Garrick M.D. (2005). DMT1 decreases metal-related injury in the lung. Am. J. Physiol.-Lung Cell. Mol. Physiol..

[77] Castellani R.J., Moreira P.I., Liu G., Dobson J., Perry G., Smith M.A., Zhu X. (2007). Iron: The Redox-active center of oxidative stress in Alzheimer disease. Neurochem. Res..

[78] Burdo J.R., Menzies S.L., Simpson I.A., Garrick L.M., Garrick M.D., Dolan K.G., Haile D.J., Beard J.L., Connor J.R. (2001). Distribution of divalent metal transporter 1 and metal transport protein 1 in the normal and Belgrade rat. J. Neurosci. Res..

[79] Burdo J.R., Simpson I.A., Menzies S., Beard J., Connor J.R. (2004). Regulation of the profile of iron-management proteins in brain microvasculature. J. Cereb. Blood Flow. Metab..

[80] Wang X.S., Ong W.Y., Connor J.R. (2001). A light and electron microscopic study of the iron transporter protein DMT-1 in the monkey cerebral neocortex and hippocampus. J. Neurocytol..

[81] Moos T., Morgan E.H. (2004). The significance of the mutated divalent metal transporter (DMT1) on iron transport into the Belgrade rat brain. J. Neurochem..

[82] Tchernitchko D., Bourgeois M., Martin M.E., Beaumont C. (2002). Expression of the two mRNA isoforms of the iron transporter Nramp2/DMTI in mice and function of the iron responsive element. Biochem. J..

[83] Georgieff M.K., Wobken J.K., Welle J., Burdo J.R., Connor J.R. (2000). Identification and localization of divalent metal transporter-1 (DMT-1) in term human placenta. Placenta.

[84] Gunshin H., Allerson C.R., Polycarpou-Schwarz M., Rofts A., Rogers J.T., Kishi F., Hentze M.W., Rouault T.A., Andrews N.C., Hediger M.A. (2001). Iron-dependent regulation of the divalent metal ion transporter. FEBS Lett..

[85] Chae B.J., Lee K.S., Hwang I., Yu J.W. (2023). Extracellular acidification augments NLRP3-mediated inflammasome signaling in macrophages. Immune Netw..

[86] Bode J.G., Albrecht U., Häussinger D., Heinrich P.C., Schaper F. (2012). Hepatic acute phase proteins--regulation by IL-6- and IL-1-type cytokines involving STAT3 and its crosstalk with NF-κB-dependent signaling. Eur. J. Cell Biol..

[87] Urrutia P., Aguirre P., Esparza A., Tapia V., Mena N.P., Arredondo M., González-Billault C., Núñez M.T. (2013). Inflammation alters the expression of DMT1, FPN1 and hepcidin, and it causes iron accumulation in central nervous system cells. J. Neurochem..

[88] You L.H., Yan C.Z., Zheng B.J., Ci Y.Z., Chang S.Y., Yu P., Gao G.F., Li H.Y., Dong T.Y., Chang Y.Z. (2017). Astrocyte hepcidin is a key factor in LPS-induced neuronal apoptosis. Cell Death Dis..

[89] Asperti M., Denardo A., Gryzik M., Persson K.E.M., Westerberg G., Öhd J., Poli M. (2024). Sevuparin strongly reduces hepcidin expression in cells, mice, and healthy human volunteers. Hemasphere.

[90] Volani C., Doerrier C., Demetz E., Haschka D., Paglia G., Lavdas A.A., Gnaiger E., Weiss G. (2017). Dietary iron loading negatively affects liver mitochondrial function. Metallomics.

[91] Galaris D., Barbouti A., Pantopoulos K. (2019). Iron homeostasis and oxidative stress: An intimate relationship. Biochim. Biophys. Acta Mol. Cell Res..

[92] Katsarou A., Pantopoulos K. (2020). Basics and principles of cellular and systemic iron homeostasis. Mol. Asp. Med..

[93] Zheng J., Conrad M. (2025). Ferroptosis: When metabolism meets cell death. Physiol. Rev..

[94] Hajjar S., Zhou X. (2023). pH sensing at the intersection of tissue homeostasis and inflammation. Trends Immunol..

[95] Tan Q., Zhang X., Li S., Liu W., Yan J., Wang S., Cui F., Li D., Li J. (2023). DMT1 differentially regulates mitochondrial complex activities to reduce glutathione loss and mitigate ferroptosis. Free Radic. Biol. Med..

[96] Riemann A., Wußling H., Loppnow H., Fu H., Reime S., Thews O. (2016). Acidosis differently modulates the inflammatory program in monocytes and macrophages. Biochim. Biophys. Acta.

[97] Erra Díaz F., Dantas E., Geffner J. (2018). Unravelling the Interplay between Extracellular Acidosis and Immune Cells. Mediators Inflamm..

[98] Rajamäki K., Nordström T., Nurmi K., Åkerman K.E., Kovanen P.T., Öörni K., Eklund K.K. (2013). Extracellular acidosis is a novel danger signal alerting innate immunity via the NLRP3 inflammasome. J. Biol. Chem..

[99] Swanson K.V., Deng M., Ting J.P. (2019). The NLRP3 inflammasome: Molecular activation and regulation to therapeutics. Nat. Rev. Immunol..

[100] Li C., Chen M., He X., Ouyang D. (2021). A mini-review on ion fluxes that regulate NLRP3 inflammasome activation. Acta Biochim. Biophys. Sin..

[101] Nakamura K., Kawakami T., Yamamoto N., Tomizawa M., Fujiwara T., Ishii T., Harigae H., Inoue K., Takahashi Y., Sato T. (2016). Activation of the NLRP3 inflammasome by cellular labile iron. Exp. Hematol..

[102] Aguree S. (2025). Iron-Inflammasome Crosstalk in Adipose Tissue: Unresolved Roles of NLRP3 and IL-1β in Metabolic Inflammation. Int. J. Mol. Sci..

[103] Luo M., Yan D., Sun Q., Tao J., Xu L., Sun H., Zhao H. (2020). Ginsenoside Rg1 attenuates cardiomyocyte apoptosis and inflammation via the TLR4/NF-kB/NLRP3 pathway. J. Cell. Biochem..

[104] Nguyen L.T.N., Nguyen H.D., Kim Y.J., Nguyen T.T., Lai T.T., Lee Y.K., Ma H.I., Kim Y.E. (2022). Role of NLRP3 inflammasome in parkinson’s disease and therapeutic considerations. J. Park. Dis..

[105] Harrison D., Billinton A., Bock M.G., Doedens J.R., Gabel C.A., Holloway M.K., Porter R.A., Reader V., Scanlon J., Schooley K. (2023). Discovery of clinical candidate NT-0796, a brain-penetrant and highly potent NLRP3 inflammasome inhibitor for neuroinflammatory disorders. J. Med. Chem..

[106] Clarke N., Thornton P., Reader V., Lindsay N., Digby Z., Mullen B., Gorman M., Jacobson E., Langdon G., Johnstone H. (2025). Anti-neuroinflammatory and anti-inflammatory effects of the NLRP3 inhibitor NT-0796 in subjects with Parkinson’s Disease. Mov. Disord..

[107] Urrutia P.J., Bórquez D.A., Núñez M.T. (2021). Inflaming the Brain with Iron. Antioxidants.

[108] Ana B. (2024). Aged-related changes in microglia and neurodegenerative diseases: Exploring the connection. Biomedicines.

[109] Si J., Guo J., Zhang X., Li W., Zhang S., Shang S., Zhang Q. (2024). Hypoxia-induced activation of HIF-1alpha/IL-1beta axis in microglia promotes glioma progression via NF-κB-mediated upregulation of heparanase expression. Biol. Direct.

[110] Zhao J., Bi W., Xiao S., Lan X., Cheng X., Zhang J., Lu D., Wei W., Wang Y., Li H. (2019). Neuroinflammation induced by lipopolysaccharide causes cognitive impairment in mice. Sci. Rep..

[111] Liang T., Yang S.X., Qian C., Du L.D., Qian Z.M., Yung W.H., Ke Y. (2024). HMGB1 mediates inflammation-induced DMT1 increase and dopaminergic neurodegeneration in the early stage of Parkinsonism. Mol. Neurobiol..

[112] Jia Y., Deng H., Qin Q., Ma Z. (2020). JWH133 inhibits MPP^+^-induced inflammatory response and iron influx in astrocytes. Neurosci. Lett..

[113] Amor S., McNamara N.B., Gerrits E., Marzin M.C., Kooistra S.M., Miron V.E., Nutma E. (2022). White matter microglia heterogeneity in the CNS. Acta Neuropathol..

[114] Armstrong R.C., Sullivan G.M., Perl D.P., Rosarda J.D., Radomski K.L. (2024). White matter damage and degeneration in traumatic brain injury. Trends Neurosci..

[115] Loane D.J., Kumar A., Stoica B.A., Cabatbat R., Faden A.I. (2014). Progressive neurodegeneration after experimental brain trauma: Association with chronic microglial activation. Neuropathol. Exp. Neurol..

[116] Jia J., Zheng L., Ye L., Chen J., Shu S., Xu S., Bao X., Xia S., Liu R., Xu Y. (2023). CD11c+ microglia promotes white matter repair after ischemic stroke. Cell Death Dis..

[117] Yan S., Sun J., Chen Y., Selim M., Lou M. (2013). Brain iron deposition in white matter hyperintensities: A 3-T MRI study. Age.

[118] McCarthy R.C., Sosa J.C., Gardeck A.M., Baez A.S., Lee C.H., Wessling-Resnick M. (2018). Inflammation-induced iron transport and metabolism by brain microglia. J. Biol. Chem..

[119] Nnah I.C., Lee C.H., Wessling-Resnick M. (2020). Iron potentiates microglial interleukin-1β secretion induced by amyloid-β. J. Neurochem..

[120] Batista A.F., Rody T., Forny-Germano L., Cerdeiro S., Bellio M., Ferreira S.T., Munoz D.P., De Felice F.G. (2021). Interleukin-1β mediates alterations in mitochondrial fusion/fission proteins and memory impairment induced by amyloid-β oligomers. J. Neuroinflamm..

[121] Robertson K.V., Rodriguez A.S., Cartailler J.P., Shrestha S., Schleh M.W., Schroeder K.R., Valenti A.M., Kramer A.T., Harrison F.E., Hasty A.H. (2024). Knockdown of microglial iron import gene, Slc11a2, worsens cognitive function and alters microglial transcriptional landscape in a sex-specific manner in the APP/PS1 model of Alzheimer’s disease. J. Neuroinflamm..

[122] Qu Y., Li N., Xu M., Zhang D., Xie J., Wang J. (2022). Estrogen Up-Regulates Iron Transporters and Iron Storage Protein Through Hypoxia Inducible Factor 1 Alpha Activation Mediated by Estrogen Receptor β and G Protein Estrogen Receptor in BV2 Microglia Cells. Neurochem. Res..

[123] Villarroel P., Le Blanc S., Arredondo M. (2012). Interleukin-6 and lipopolysaccharide modulate hepcidin mRNA expression by HepG2 cells. Biol. Trace Elem. Res..

[124] Wu L.J., Leenders A.G., Cooperman S., Meyron-Holtz E., Smith S., Land W., Tsai R.Y., Berger U.V., Sheng Z.H., Rouault T.A. (2004). Expression of the iron transporter ferroportin in synaptic vesicles and the blood-brain barrier. Brain Res..

[125] Skjørringe T., Burkhart A., Johnsen K.B., Moos T. (2015). Divalent metal transporter 1 (DMT1) in the brain: Implications for a role in iron transport at the blood-brain barrier, and neuronal and glial pathology. Front. Mol. Neurosci..

[126] Sternberg Z., Hu Z., Sternberg D., Waseh S., Quinn J.F., Wild K., Jeffrey K., Zhao L., Garrick M. (2017). Serum hepcidin levels, iron dyshomeostasis and cognitive loss in Alzheimer’s Disease. Aging Dis..

[127] Andersson E., Schultz N., Saito T., Saido T.C., Blennow K., Gouras G.K., Zetterberg H., Hansson O. (2023). Cerebral Abeta deposition precedes reduced cerebrospinal fluid and serum Abeta42/Abeta40 ratios in the App(NL-F/NL-F) knock-in mouse model of Alzheimer’s disease. Alzheimer’s Res. Ther..

[128] Castellazzi M., Patergnani S., Donadio M., Giorgi C., Bonora M., Bosi C., Brombo G., Pugliatti M., Seripa D., Zuliani G. (2019). Autophagy and mitophagy biomarkers are reduced in sera of patients with Alzheimer’s disease and mild cognitive impairment. Sci. Rep..

[129] Cheng J., Liao Y., Dong Y., Hu H., Yang N., Kong X., Li S., Li X., Guo J., Qin L. (2020). Microglial autophagy defect causes Parkinson Disease-like symptoms by accelerating inflammasome activation in mice. Autophagy.

[130] Baiguera C., Alghisi M., Pinna A., Bellucci A., De Luca M.A., Frau L., Morelli M., Ingrassia R., Benarese M., Porrini V. (2012). Late-onset Parkinsonism in NFκB/c-Rel-deficient mice. Brain.

[131] Salazar J., Mena N., Hunot S., Prigent A., Alvarez-Fischer D., Arredondo M., Duyckaerts C., Sazdovitch V., Zhao L., Garrick L.M. (2008). Divalent metal transporter 1 (DMT1) contributes to neurodegeneration in animal models of Parkinson’s disease. Proc. Natl. Acad. Sci. USA.

[132] Roth J.A., Singleton S., Feng J., Garrick M., Paradkar P.N. (2010). Parkin regulates metal transport via proteasomal degradation of the 1B isoforms of divalent metal transporter 1. J. Neurochem..

[133] Ben-Shachar D., Youdim M.B. (1991). Intranigral iron injection induces behavioural and biochemical parkinsonism in rats. J. Neurochem..

[134] Ben-Shachar D., Eshel G., Riederer P., Youdim M.B. (1992). Role of iron and iron chelation in dopaminergic-induced neurodegeneration: Implication for Parkinson’s disease. Ann. Neurol..

[135] Ben-Shachar D., Eshel G., Finberg J.P., Youdim M.B. (1991). The iron chelator desferrioxamine (Desferal) retards 6-hydroxydopamine-induced degeneration of nigrostriatal dopamine neurons. J. Neurochem..

[136] Cabantchik Z.I., Munnich A., Youdim M.B., Devos D. (2013). Regional siderosis: A new challenge for iron chelation therapy. Front. Pharmacol..

[137] Devos D., Moreau C., Devedjian J.C., Kluza J., Petrault M., Laloux C., Jonneaux A., Ryckewaert G., Garçon G., Rouaix N. (2014). Targeting chelatable iron as a therapeutic modality in Parkinson’s disease. Antioxid. Redox Signal..

[138] Levi S., Volonté M.A. (2023). Iron chelation in early Parkinson’s disease. Lancet Neurol..

[139] Ingrassia R., Garavaglia B., Memo M. (2019). DMT1 Expression and iron levels at the crossroads between aging and neurodegeneration. Front. Neurosci..

[140] Rehncrona S. (1985). Brain acidosis. Ann. Emerg. Med..

[141] Hu X., Bao Y., Li M., Zhang W., Chen C. (2024). The role of ferroptosis and its mechanism in ischemic stroke. Exp. Neurol..

[142] Chen L., Hambright W.S., Na R., Ran Q. (2015). Ablation of the Ferroptosis Inhibitor Glutathione Peroxidase 4 in Neurons Results in Rapid Motor Neuron Degeneration and Paralysis. J. Biol. Chem..

[143] Lin Y.H., Wang X.W., Li Y.A., Chen T.Y., Lian W.S., Wang F.S., Lan M.Y., Liou C.W., Lin T.K. (2026). Dysregulated iron homeostasis Drives mitochondrial Injury and ferroptosis susceptibility in MELAS fibroblasts. Mitochondrion.

[144] Lorenz S.M., Wahida A., Bostock M.J., Seibt T., Santos Dias Mourão A., Levkina A., Trümbach D., Soudy M., Emler D., Rothammer N. (2026). A fin-loop-like structure in GPX4 underlies neuroprotection from ferroptosis. Cell.

[145] Pelizzoni I., Zacchetti D., Campanella A., Grohovaz F., Codazzi F. (2013). Iron uptake in quiescent and inflammation-activated astrocytes: A potentially neuroprotective control of iron burden. Biochim. Biophys. Acta.

[146] Salami A., Papenberg G., Sitnikov R., Laukka E.J., Persson J., Kalpouzos G. (2021). Elevated neuroinflammation contributes to the deleterious impact of iron overload on brain function in aging. Neuroimage.

[147] Huang E., Ong W.Y., Go M.L., Connor J.R. (2006). Upregulation of iron regulatory proteins and divalent metal transporter-1 isoforms in the rat hippocampus after kainate induced neuronal injury. Exp. Brain Res..

[148] Kruer M.C., Boddaert N., Schneider S.A., Houlden H., Bhatia K.P., Gregory A., Anderson J.C., Rooney W.D., Hogarth P., Hayflick S.J. (2012). Neuroimaging features of neurodegeneration with brain iron accumulation. AJNR Am. J. Neuroradiol..

[149] Gregory A., Kurian M.A., Wilson J., Hayflick S., Adam M.P., Bick S., Mirzaa G.M., Pagon R.A., Wallace S.E., Amemiya A. (2013). Neurodegeneration with brain iron accumulation disorders overview. Gene Reviews.

[150] Arber C.E., Li A., Houlden H., Wray S. (2016). Insights into molecular mechanisms of disease in neurodegeneration with brain iron accumulation: Unifying theories. Neuropathol. Appl. Neurobiol..

[151] Levi S., Tiranti V. (2019). Neurodegeneration with brain iron accumulation disorders: Valuable models aimed at understanding the pathogenesis of iron deposition. Pharmaceuticals.

[152] Levi S., Rovida E. (2015). Neuroferritinopathy: From ferritin structure modification to pathogenetic mechanism. Neurobiol. Dis..

[153] Kassubek R., Uttner I., Schönfeldt-Lecuona C., Kassubek J., Connemann B.J. (2017). Extending the aceruloplasminemia phenotype: NBIA on imaging and acanthocytosis, yet only minor neurological findings. J. Neurol. Sci..

[154] Wilson J.L., Allison Gregory A., Kurian M.A., Bushlin I., Mochel F., Emrick L., Adang L., Hogarth P., Hayflick S.J., BPAN Guideline Contributing Author Group (2021). Consensus clinical management guideline for beta-propeller protein-associated neurodegeneration. Dev. Med. Child. Neurol..

[155] Russo C., Ardissone A., Freri E., Gasperini S., Moscatelli M., Zorzi G., Panteghini C., Castellotti B., Garavaglia B., Nardocci N. (2018). Substantia Nigra swelling and dentate nucleus T2 hyperintensity may be early magnetic resonance imaging signs of β-propeller protein-associated neurodegeneration. Mov. Disord. Clin. Pract..

[156] Manti F., Panteghini C., Garavaglia B., Leuzzi V. (2021). Neurodevelopmental disorder and late-onset degenerative Parkinsonism in a patient with a WDR45 defect. Mov. Disord. Clin. Pract..

[157] Woltjer R.L., Reese L.C., Richardson B.E., Tran H., Green S., Pham T., Chalupsky M., Gabriel I., Light T., Sanford L. (2015). Pallidal neuronal apolipoprotein E in pantothenate kinase-associated neurodegeneration recapitulates ischemic injury to the globus pallidus. Mol. Genet. Metab..

[158] Yang L., Zhang B., Yin L., Cai B., Shan H., Zhang L., Lu Y., Bi Z. (2011). Tanshinone IIA prevented brain iron dyshomeostasis in cerebral ischemic rats. Cell. Physiol. Biochem..

[159] Saitsu H., Nishimura T., Muramatsu K., Kodera H., Kumada S., Sugai K., Kasai-Yoshida E., Sawaura N., Nishida H., Hoshino A. (2013). De novo mutations in the autophagy gene WDR45 cause static encephalopathy of childhood with neurodegeneration in adulthood. Nat. Genet..

[160] Ingrassia R., Memo M., Garavaglia B. (2017). Ferrous iron up-regulation in fibroblasts of patients with beta propeller protein-associated neurodegeneration (BPAN). Front. Genet..

[161] Toth H.L., Greenbaum L.A. (2003). Severe acidosis caused by starvation and stress. Am. J. Kidney Dis..

[162] Aring L., Choi E.K., Kopera H., Lanigan T., Iwase S., Klionsky D.J., Seo Y.A. (2022). A neurodegeneration gene, WDR45, links impaired ferritinophagy to iron accumulation. J. Neurochem..

[163] Lee M., Yoon J.H. (2015). Metabolic interplay between glycolysis and mitochondrial oxidation: The reverse Warburg effect and its therapeutic implication. World J. Biol. Chem..

[164] Zhu Y., Fujimaki M., Snape L., Lopez A., Fleming A., Rubinsztein D.C. (2024). Loss of WIPI4 in neurodegeneration causes autophagy-independent ferroptosis. Nat. Cell Biol..

[165] Ordway B., Gillies R.J., Damaghi M. (2021). Extracellular acidification induces lysosomal dysregulation. Cells.

[166] Garavaglia B., Nasca A., Mitola S., Ingrassia R. (2024). WDR45-dependent impairment of cell cycle in fibroblasts of patients with beta propeller protein-associated neurodegeneration (BPAN). Biochim. Biophys. Acta Mol. Cell Res..

[167] Slade L., Biswas D., Kienesberger P.C., Pulinilkunnil T. (2022). Loss of transcription factor EB dysregulates the G1/S transition and DNA replication in mammary epithelial cells. J. Biol. Chem..

[168] Chen L., Ma Y., Ma X., Liu L., Jv X., Li A., Shen Q., Jia W., Qu L., Shi L. (2023). TFEB regulates cellular labile iron and prevents ferroptosis in a TfR1-dependent manner. Free Radic. Biol. Med..

[169] Losby M., Hayes M., Valfort A., Sopariwala D.H., Sanders R., Walker J.K., Xu W., Narkar V.A., Zhang L., Billon C. (2024). The Estrogen Receptor-Related Orphan Receptors Regulate Autophagy through TFEB. Mol. Pharmacol..

[170] Hunt J.F., Fang K., Malik R., Snyder A., Malhotra N., Platts-Mills T.A., Gaston B. (2000). Endogenous airway acidification. Implications for asthma pathophysiology. Am. J. Respir. Crit. Care Med..

[171] Kostikas K., Papatheodorou G., Ganas K., Psathakis K., Panagou P., Loukides S. (2002). pH in expired breath condensate of patients with inflammatory airway diseases. Am. J. Respir. Crit. Care Med..

[172] Tate S., MacGregor G., Davis M., Innes J.A., Greening A.P. (2002). Airways in cystic fibrosis are acidified: Detection by exhaled breath condensate. Thorax.

[173] Borrill Z., Starkey C., Vestbo J., Singh D. (2005). Reproducibility of exhaled breath condensate pH in chronic obstructive pulmonary disease. Eur. Respir. J..

[174] Sandmeier P., Speich R., Grebski E., Vogt P., Russi E.W., Weder W., Boehler A. (2005). Iron accumulation in lung allografts is associated with acute rejection but not with adverse outcome. Chest.

[175] Dupont L.J., Dewandeleer Y., Vanaudenaerde B.M., Van Raemdonck D.E., Verleden G.M. (2006). The pH of exhaled breath condensate of patients with allograft rejection after lung transplantation. Am. J. Transplant..

[176] MacNee W., Rennard S.I., Hunt J.F., Edwards L.D., Miller B.E., Locantore N.W., Tal-Singer R. (2011). Evaluation of exhaled breath condensate pH as a biomarker for COPD. Respir. Med..

[177] Ghio A.J. (2016). Asthma as a disruption in iron homeostasis. Biometals.

[178] Ghio A.J., Soukup J.M., McGee J., Madden M.C., Esther C.R. (2018). Iron concentration in exhaled breath condensate decreases in ever-smokers and COPD patients. J. Breath Res..

[179] Ali M.K., Kim R.Y., Brown A.C., Mayall J.R., Karim R., Pinkerton J.W., Liu G., Martin K.L., Starkey M.R., Pillar A.L. (2020). Crucial role for lung iron level and regulation in the pathogenesis and severity of asthma. Eur. Respir. J..

[180] Ali M.K., Kim R.Y., Brown A.C., Donovan C., Vanka K.S., Mayall J.R., Liu G., Pillar A.L., Jones-Freeman B., Xenaki D. (2020). Critical role for iron accumulation in the pathogenesis of fibrotic lung disease. J. Pathol..

[181] Gifford A.H., Polineni D., He J., D’Amico J.L., Dorman D.B., Williams M.A., Nymon A.B., Balwan A., Budden T., Zuckerman J.B. (2021). A pilot study of cystic fibrosis exacerbation response phenotypes reveals contrasting serum and sputum iron trends. Sci. Rep..

[182] van der Sar I.G., Wijsenbeek M.S., Moor C.C. (2023). Exhaled breath analysis in interstitial lung disease. Curr. Opin. Pulm. Med..

[183] Vaughan J., Ngamtrakulpanit L., Pajewski T.N., Turner R., Nguyen T.A., Smith A., Urban P., Hom S., Gaston B., Hunt J. (2003). Exhaled breath condensate pH is a robust and reproducible assay of airway acidity. Eur. Respir. J..

[184] Do R., Bartlett K.H., Chu W., Dimich-Ward H., Kennedy S.M. (2008). Within- and between-person variability of exhaled breath condensate pH and NH4+ in never and current smokers. Respir. Med..

[185] Hoffmeyer F., Harth V., Bunger J., Bruning T., Raulf-Heimsoth M. (2009). Leukotriene B4, 8-iso-prostaglandin F2 alpha, and pH in exhaled breath condensate from asymptomatic smokers. J. Physiol. Pharmacol..

[186] Zhang W.Z., Butler J.J., Cloonan S.M. (2019). Smoking-induced iron dysregulation in the lung. Free Radic. Biol. Med..

[187] Ghio A.J., Pavlisko E.N., Roggli V.L., Todd N.W., Sangani R.G. (2022). Cigarette smoke particle-induced lung injury and iron homeostasis. Int. J. Chronic Obstr. Pulm. Dis..

[188] Ghio A.J., Carter J.D., Richards J.H., Richer L.D., Grissom C.K., Elstad M.R. (2003). Iron and iron-related proteins in the lower respiratory tract of patients with acute respiratory distress syndrome. Crit. Care Med..

[189] Nguyen N.B., Callaghan K.D., Ghio A.J., Haile D.J., Yang F. (2006). Hepcidin expression and iron transport in alveolar macrophages. Am. J. Physiol. Lung Cell. Mol. Physiol..

[190] Kim J., Molina R.M., Donaghey T.C., Buckett P.D., Brain J.D., Wessling-Resnick M. (2011). Influence of DMT1 and iron status on inflammatory responses in the lung. Am. J. Physiol. Lung Cell. Mol. Physiol..

[191] Ghio A.J., Roggli V.L., Soukup J.M., Richards J.H., Randell S.H., Muhlebach M.S. (2013). Iron accumulates in the lavage and explanted lungs of cystic fibrosis patients. J. Cyst. Fibros..

[192] Maciel A.T., Noritomi D.T., Park M. (2010). Metabolic acidosis in sepsis. Endocr. Metab. Immune Disord. Drug Targets.

[193] Suetrong B., Walley K.R. (2016). Lactic acidosis in sepsis: It’s not all anaerobic: Implications for diagnosis and management. Chest.

[194] Ghio C., Soukup J.M., Dailey L.A., Ghio A.J., Schreinemachers D.M., Koppes R.A., Koppes A.N. (2022). Lactate Production can Function to Increase Human Epithelial Cell Iron Concentration. Cell. Mol. Bioeng..

[195] Rapola J., Heikkilä P., Fellman V. (2002). Pathology of lethal fetal growth retardation syndrome with aminoaciduria, iron overload, and lactic acidosis (GRACILE). Pediatr. Pathol. Mol. Med..

[196] Tebbi C.K., Steffensen T.S., Thorkelsson T., Gudmundsson J.A., Gilbert-Barness E. (2011). Clinicopathologic conference: Multiple fetal demises, lactic acidosis and hepatic iron accumulation. Fetal Pediatr. Pathol..

[197] Zhang S., Liu W., Ganz T., Liu S. (2024). Exploring the relationship between hyperlactatemia and anemia. Trends Endocrinol. Metab..

[198] Artis W.M., Fountain J.A., Delcher H.K., Jones H.E. (1982). A mechanism of susceptibility to mucormycosis in diabetic ketoacidosis: Transferrin and iron availability. Diabetes.

[199] Lee J.U., Hong J., Shin H., Ryu C.B., Park S.W., Jeong S.H. (2022). Overexpression of V-ATPase B2 attenuates lung injury/fibrosis by stabilizing lysosomal membrane permeabilization and increasing collagen degradation. Exp. Mol. Med..

[200] McAlinden K.D., Kota A., Haghi M., Ghavami S., Sharma P. (2020). Pharmacologic Inhibition of Vacuolar H(+)ATPase Attenuates Features of Severe Asthma in Mice. Am. J. Respir. Cell Mol. Biol..

[201] Capparelli R., Palumbo D., Iannaccone M., Iannelli D. (2009). Human V-ATPase gene can protect or predispose the host to pulmonary tuberculosis. Genes Immun..

[202] Nishisho T., Hata K., Nakanishi M., Morita Y., Sun-Wada G.H., Wada Y., Yasui N., Yoneda T. (2011). The a3 isoform vacuolar type H+-ATPase promotes distant metastasis in the mouse B16 melanoma cells. Mol. Cancer Res..

[203] Lu Q., Lu S., Huang L., Wang T., Wan Y., Zhou C.X., Zhang C., Zhang Z., Li X. (2013). The expression of V-ATPase is associated with drug resistance and pathology of non-small-cell lung cancer. Diagn. Pathol..

[204] Kong X.M., Zhang G.H., Huo Y.K., Zhao X.H., Cao D.W., Guo S.F., Li A.M., Zhang X.R. (2015). MicroRNA-140-3p inhibits proliferation, migration and invasion of lung cancer cells by targeting ATP6AP2. Int. J. Clin. Exp. Pathol..

[205] Jin H.O., Hong S.E., Kim C.S., Park J.A., Kim J.H., Kim J.Y., Kim B., Chang Y.H., Hong S.I., Hong Y.J. (2015). Combined effects of EGFR tyrosine kinase inhibitors and vATPase inhibitors in NSCLC cells. Toxicol. Appl. Pharmacol..

[206] Grillo A.S., SantaMaria A.M., Kafina M.D., Cioffi A.G., Huston N.C., Han M., Seo Y.A., Yien Y.Y., Nardone C., Menon A.V. (2017). Restored iron transport by a small molecule promotes absorption and hemoglobinization in animals. Science.

[207] Garrick M.D., Garrick L.M., Zhao L., Collins J.F., Soukup J., Ghio A.J. (2019). A direct comparison of divalent metal-ion transporter (DMT1) and hinokitiol, a potential small molecule replacement. Biometals.

[208] Müller A., Cadenas E., Graf P., Sies H. (1984). A novel biologically active seleno-organic compound--I. Glutathione peroxidase-like activity in vitro and antioxidant capacity of PZ 51 (Ebselen). Biochem. Pharmacol..

[209] Paernham M.J., Sies H. (2013). The early research and development of ebselen. Biochem. Pharmacol..

[210] Yamaguchi T., Sano K., Takakura K., Saito I., Shinohara Y., Asano T., Yasuhara H. (1998). Ebselen in acute ischemic stroke: A placebo-controlled, double-blind clinical trial. Stroke.

[211] Ogawa A., Yoshimoto T., Kikuchi H., Sano K., Saito I., Yamaguchi T., Yasuhara H. (1999). Ebselen in acute middle cerebral artery occlusion: A placebo-controlled, double-blind clinical trial. Cerebrovasc. Dis..

[212] Wetli H.A., Buckett P.D., Wessling-Resnick M. (2006). Small-molecule screening identifies the selanazal drug ebselen as a potent inhibitor of DMT1-mediated iron uptake. Chem. Biol..

[213] Klann I.P., Martini F., Gonçalves Rosa S., Nogueira C.W. (2020). Ebselen reversed peripheral oxidative stress induced by a mouse model of sporadic Alzheimer’s disease. Mol. Biol. Rep..

[214] Ghazaiean M., Aliasgharian A., Karami H., Darvishi-Khezri H. (2023). Ebselen: A promising therapy protecting cardiomyocytes from excess iron in iron-overloaded thalassemia patients. Open Med..

[215] Mukem S., Sayoh I., Maungchanburi S., Thongbuakaew T. (2023). Ebselen, iron uptake inhibitor, alleviates iron overload-induced senescence-like neuronal cells SH-SY5Y via suppressing the mTORC1 signaling pathway. Adv. Pharmacol. Pharm. Sci..

[216] Wang X., Zhang M., Flores S.R.L., Woloshun R.R., Yang C., Yin L., Xiang P., Xu X., Garrick M.D., Vidyasagar S. (2019). Oral gavage of ginger nanoparticle-derived lipid vectors carrying Dmt1 siRNA blunts iron loading in murine hereditary hemochromatosis. Mol. Ther..

[217] Wang X., Zhang M., Woloshun R.R., Yu Y., Lee J.K., Flores S.R.L., Merlin D., Collins J.F. (2021). Oral administration of ginger-derived lipid nanoparticles and Dmt1 siRNA potentiates the effect of dietary iron restriction and mitigates pre-existing iron overload in Hamp KO mice. Nutrients.

[218] Devos D., Labreuche J., Rascol O., Corvol J.C., Duhamel A., Guyon Delannoy P., Poewe W., Compta Y., Pavese N., Růžička E. (2022). Trial of Deferiprone in Parkinson’s Disease. N. Engl. J. Med..

[219] Ayton S., Moreau C., Devos D., Bush A.I. (2025). Iron on trial: Recasting the role of iron in neurodegeneration. Brain.

[220] Ayton S., Barton D., Brew B., Brodtmann A., Clarnette R., Desmond P., Devos D., Ellis K.A., Fazlollahi A., Fradette C. (2025). Deferiprone in Alzheimer Disease: A Randomized Clinical Trial. JAMA Neurol..

[221] Bulli I., Dettori I., Coppi E., Cherchi F., Venturini M., Di Cesare Mannelli L., Ghelardini C., Nocentini A., Supuran C.T., Pugliese A.M. (2021). Role of carbonic anhydrase in cerebral ischemia and carbonic anhydrase inhibitors as putative protective agents. Int. J. Mol. Sci..

[222] Supuran C.T. (2018). Carbonic anhydrase inhibitors and their potential in a range of therapeutic areas. Expert Opin. Ther. Pat..

[223] Coll R.C., Schroder K. (2025). Inflammasome components as new therapeutic targets in inflammatory disease. Nat. Rev. Immunol..

[224] Cabral J.E., Wu A., Zhou H., Pham M.A., Lin S., McNulty R. (2025). Targeting the NLRP3 inflammasome for inflammatory disease therapy. Trends Pharmacol. Sci..

[225] Lu L., Jifu C., Xia J., Wang J. (2024). E3 ligases and DUBs target ferroptosis: A potential therapeutic strategy for neurodegenerative diseases. Biomed. Pharmacother..

[226] Ingrassia R., Garrick M.D. (2024). Iron overload accompanying extracellular acidosis and neurodegeneration. Neurosci. Neurol..

